# The role of artificial intelligence in analysis of biofluid markers for diagnosis and management of glaucoma: A systematic review

**DOI:** 10.1177/11206721221140948

**Published:** 2022-11-25

**Authors:** Aidan Pucchio, Saffire Krance, Daiana R Pur, Arshpreet Bassi, Rafael Miranda, Tina Felfeli

**Affiliations:** 1 School of Medicine, 4257 Queen's University, Kingston, Ontario, Canada; 2 70384 Schulich School of Medicine and Dentistry, Western University, London, Ontario, Canada; 3 Toronto Health Economics and Technology Assessment Collaborative, 7938 University of Toronto, Toronto, Ontario, Canada; 4 Department of Ophthalmology and Visual Sciences, 7938 University of Toronto, Toronto, Ontario, Canada; 5 The Institute of Health Policy, Management and Evaluation, 7938 University of Toronto, Toronto, Ontario, Canada

**Keywords:** artificial intelligence, biofluid, glaucoma, diagnosis, pathogenesis

## Abstract

**Purpose:**

This review focuses on utility of artificial intelligence (AI) in analysis of biofluid markers in glaucoma. We detail the accuracy and validity of AI in the exploration of biomarkers to provide insight into glaucoma pathogenesis.

**Methods:**

A comprehensive search was conducted across five electronic databases including Embase, Medline, Cochrane Central Register of Controlled Trials, Cochrane Database of Systematic Reviews, and Web of Science. Studies pertaining to biofluid marker analysis using AI or bioinformatics in glaucoma were included. Identified studies were critically appraised and assessed for risk of bias using the Joanna Briggs Institute Critical Appraisal tools.

**Results:**

A total of 10,258 studies were screened and 39 studies met the inclusion criteria, including 23 cross-sectional studies (59%), nine prospective cohort studies (23%), six retrospective cohort studies (15%), and one case-control study (3%). Primary open angle glaucoma (POAG) was the most commonly studied subtype (55% of included studies). Twenty-four studies examined disease characteristics, 10 explored treatment decisions, and 5 provided diagnostic clarification. While studies examined at entire metabolomic or proteomic profiles to determine changes in POAG, there was heterogeneity in the data with over 175 unique, differentially expressed biomarkers reported. Discriminant analysis and artificial neural network predictive models displayed strong differentiating ability between glaucoma patients and controls, although these tools were untested in a clinical context.

**Conclusion:**

The use of AI models could inform glaucoma diagnosis with high sensitivity and specificity. While insight into differentially expressed biomarkers is valuable in pathogenic exploration, no clear pathogenic mechanism in glaucoma has emerged.

## Introduction

Glaucoma is the leading cause of irreversible blindness worldwide, with a projected prevalence of 111.8 million by 2040.^1,2^ With the growing burden of glaucoma, accurate and timely solutions to address disparities in screening, diagnosis, and management are critical.^1,2^ Novel advances in glaucoma research and the development of clinical tools with the use of biomarkers have shown promise in enhancing patient care.^3–7^ Molecular etiologies such as cytokine and immunologic dysregulation, lipid metabolism abnormalities, lysosomal action, angiogenesis, and metabolic syndromes have enabled exploration of the pathogenesis and mechanisms of glaucoma development.^7^ These biomarkers are often contained in biofluids such as serum, tears, aqueous humour, and vitreous humour, which may be obtained in clinical and surgical contexts.^8,9^ Given the complexity of interactions between various biomarkers and their relationship with a multitude of clinical characteristics, advanced strategies are required to uncover meaningful trends for paradigm shift in the field of research and more personalized glaucoma management strategies.

Artificial intelligence (AI) has robust and varied applications in glaucoma care for improving efficiency, with preliminary applications that display strong diagnostic and prognostic performance.^3,4,10^ Supervised AI techniques such as artificial neural networks (ANN) or discriminant analysis are trained using defined cases and can learn to classify data or predict outcomes.^11–16^ In contrast to supervised AI, unsupervised AI including cluster analysis and principal component analysis (PCA) is adept at determining trends in highly dimensional data, with unsupervised tools being used to group unlabeled data based on similarities or differences and find associations between variables in large data sets.^11–16^ Bioinformatics applications such as pathway analysis or Kyoto Encyclopedia of Genes and Genomes (KEGG) can translate these complex findings into interpretable information. All of these techniques have been implicated in diagnosis, monitoring glaucomatous progression, treatment selection, and differentiation between glaucoma and other ophthalmic conditions.^17–24^ Traditional imaging focused AI applications have rivaled the diagnostic ability of trained ophthalmologists in glaucoma diagnosis using optical coherence tomography (OCT) or fundoscopy.^17–24^ In more recent research efforts, biofluid marker analysis using AI is being investigated to develop more complex and complete clinical tools that may serve as point of care diagnostic tools, determination of underlying glaucoma etiology, and prediction of glaucomatous progression.^25–27^ Clinical tools using AI could allow for automated glaucoma screening at primary care facilities or allied eye care providers, leading to improved patient outcomes and efficient use of specialist time and resources.^1,2^ Biofluid marker analysis using AI could augment traditional diagnostic and prognostic clinical tool development.^25–29^ Further, exploration of these biomarkers could enable improved understanding of disease pathogenesis and the subsequent development of novel treatments.^28^ Herein we aim to systematically review the available literature and describe the application of AI and bioinformatics in the analysis of biofluid markers in glaucoma. This study will provide a detailed analysis of the AI and bioinformatics tools used in the study of glaucoma and the goals of their application, appraise the available evidence for clinical implementation of these technologies, and identify areas for future studies.

## Methods

This systematic review was conducted in accordance with the Preferred Reporting Items for a Systematic Review and Meta-analysis (PRISMA) guidelines.^30^ The protocol was registered in PROSPERO (reg. CRD42020196749). Since this is a systematic review of published studies and does not involve human subjects, an ethics approval from our Institutional Review Board was not required. This systematic review is part of a series of systematic reviews on AI/bioinformatic analysis of biofluid markers in ophthalmology, including systematic reviews of AI/bioinformatic analysis of biofluid markers in age-related macular degeneration, retinal occlusive disease, and uveal disease. These additional conditions will be reported elsewhere.

### Search strategy

The search strategy was developed in consultation with an experienced librarian. A comprehensive search was conducted across five electronic databases (Embase, Medline, Cochrane Central Register of Controlled Trials, Cochrane Database of Systematic Reviews, and Web of Science) for all articles meeting the inclusion and exclusion criteria from inception to August 11, 2020, and was updated on August 1, 2021. To ensure search sensitivity, the formal search used both controlled vocabulary terms and synonymous free-text words to capture the concepts of “ophthalmology” and “AI/bioinformatics” and “proteomics, metabolomics, lipidomics.” No language or study design restrictions were placed on the search. The full search strategy for all databases can be found in Supplemental Materials 1. Furthermore, hand searching of references of the included studies for relevant articles which may have not been captured in the search was performed. Grey literature indexed in Embase was captured in the search

### Selection criteria

Included studies met all of the following inclusion criteria: (1) original peer-reviewed studies that analyzed biomarker concentrations to predict or modify patient therapy or outcome/diagnosis in glaucoma or elevated intraocular pressure; (2) biomarker analysis utilized any type of AI and/or bioinformatics approaches; (3) biomarker samples were from vitreous fluid, aqueous fluid, tear fluid, plasma, serum, or ophthalmic biopsies and analyzed a protein, lipid, or metabolite. Note that studies that combined biofluid biomarkers with other types of biomarkers (e.g., imaging, genomics/transcriptomics) in their statistical models were included. Studies were excluded if they met any of the following exclusion criteria: (1) articles studying ophthalmic diseases that only affect pediatric patients (e.g., retinopathy of prematurity); (2) studies on non-human subjects (animal or cell studies); (3) studies exclusively utilizing post-mortem samples from eyes; (4) Studies not published in the English language; (5) abstracts, reviews, systematic reviews, and meta-analyses; (6) studies using only regression analysis that were cross-sectional or did not apply their findings to change treatment or predict prognosis in the study populations.

### Data collection and extraction

Abstracts and titles and subsequent full-text review were screened by two independent reviewers. Disagreements between the reviewers were resolved and adjudicated by a third reviewer. Data extraction was performed by one reviewer using standardized data collection forms. As a part of a quality check, 10% of the extractions were verified by a second independent reviewer to ensure agreement and consistency between data extractors. Key data extracted from each article included study population demographics, biofluid biomarker characterization and significance, the AI/bioinformatics tools used in analysis, and the rationale for AI/bioinformatics tool selection.

### Risk of bias assessment

The Joanna Briggs Institute Critical Appraisal Tools (Faculty of Health and Medical Sciences at the University of Adelaide, South Australia) were used to assess study risk of bias and quality.^31^ Study risk of bias was assessed by one reviewer. As a part of quality check, 10% of the risk of bias assessments were verified by a second, independent reviewer to ensure consistency between assessors. Articles were considered high risk of bias if the had <49% of questions scored yes, moderate risk of bias if they scored 50–79% of questions as yes, and low risk of bias if >80% of questions scored yes.^32^

### Synthesis of evidence

Narrative synthesis of evidence was undertaken for all included studies. Meta-analytic methods were not employed given the heterogeneity of study designs and the AI tools used. The results detailed the proportions of study type and glaucoma subtypes examined. We synthesized the accuracy of AI predictive models and explore the common applications of each type of AI. Additionally, the biomarkers and pathways that are implicated in development of each glaucoma subtype were detailed.

Data regarding the AI or bioinformatic analysis of biofluid markers in glaucoma were categorized according to study objectives and type of AI methodologies utilized. Studies were classified based on study purpose into 1) Identifying Disease Characteristics; 2) Diagnostic Clarification; and 3) Treatment Decisions. Studies focused on Identifying Disease Characteristics explored biomarkers with the intention of exploring the pathogenic mechanism of glaucoma. Studies classified under Diagnostic Clarification sought biomarkers that could differentiate glaucoma status and subtype for the purposes of diagnostic tool development. Lastly, amongst the Treatment Decisions studies, classification of biomarkers were used to guide selection of treatment options, predict outcomes following treatment selection, or inform prognosis.

## Results

### Study characteristics

**Figure 1.**
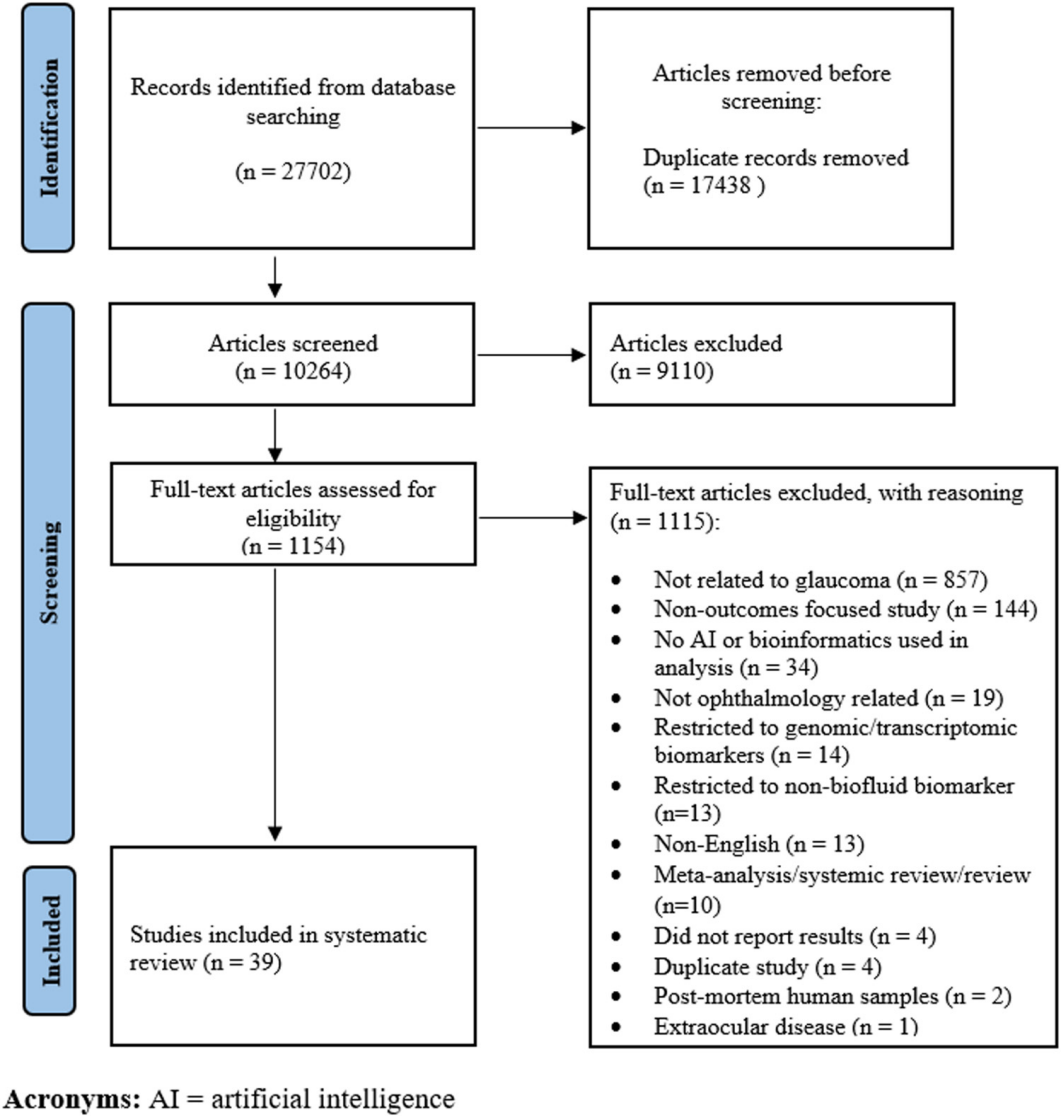
PRISMA flowchart diagram for study identification and selection.

The search strategy retrieved a total of 10,258 studies, and after removing duplicates, 39 studies met the inclusion criteria for this review (Figure 1).

Study designs included 23 cross-sectional studies (59%), nine prospective cohort studies (23%), six retrospective cohort studies (15%), and one case-control study (3%; Table 1). Studies were globally distributed, with the majority conducted in China (31%), Japan (15%), Germany (15%), and the United States (10%). Twenty-four studies examined identifying disease characteristics, 10 explored treatment decisions, and five provided diagnostic clarification. Primary open angle glaucoma (POAG) was the most commonly studied subtype in 55% of included studies, with primary angle closure glaucoma (PACG) studied in 17%, neovascular glaucoma (NVG) in 12%, and normal tension glaucoma (NTG) in 10%. Table 1 summarizes study characteristics and significant findings, with additional study characteristics summarized in Supplemental Materials 2.

**Table 1.** Summary characteristics of included studies.

First Author, Publication year	Study Design	Glaucoma type (other diseases studied)	Country of Publication	Sample Size	Classes of AI	Statistical, AI, bioinformatics Methods	Biofluid	Biomarker(s) analyzed	Significant biomarker(s) and key pathways
**Identifying Disease Characteristics**									
Adav, 2019^28^	Cross-sectional	PACG	Singapore	5 (2 cases, 3 cataract controls)	2	Unsupervised: hierarchical clustering (Gene Pattern) Bioinformatics: GO	Aqueous humor	Proteomic profile	773 proteins were differentially expressed (501 up-regulated, 272 down-regulated). Platelet degranulation, dysregulation of endocytic, exocytosis, secretion mechanisms, immune system components, oxygen homeostasis, extracellular membrane dynamics
Anton Apreutesei, 2018^25^	Retrospective cohort	POAG (diabetes)	Romania	52	1	Supervised: ANN - 1) feed forward neural network (multilayer perceptron), 2) Jordan Elman Network (JEN) type	Serum	HbA1c, glycemic level	N/A
Beutgen, 2021^33^	Cross-sectional	POAG, NTG, PXG	Germany	165 (43 POAG, 45 PXG, 31 NTG, 46 non-glaucomatous controls)	1	Bioinformatics: GO	Serum	Serological antibody profile	HSPA1A, HSPD1, YWHAZ, VDAC2, PGAM1, ENO2 (from mRNA processing, protein folding, blood coagulation and apoptosis pathways)
Buisset, 2019^34^	Cross-sectional	POAG	France	52 (26 cases, 26 cataract controls)	3	Supervised: PLS-DA Unsupervised: PCA Statistical method: univariate and multivariate regression	Aqueous humor	Metabolomic profile	Decreased taurine and spermine, increased concentrations of creatinine, carnitine, 3 short-chain acylcarnitines, 7 amino acids, 7 phosphatidylcholines, 1 lysophosphatidylcholine, 1 sphingomyelin.
Goto, 2013^35^	Retrospective cohort	NVG (PDR, diabetes)	Japan	512	1	Statistical method: Cox proportional hazards regression model	Serum	HbA1c, creatinine concentration	None.
Grus, 2008^36^	Cross-sectional	POAG	Germany	107 (52 cases, cataract 55 controls)	1	Supervised: DA, ANN (unspecified type)	Aqueous humor	Proteomic profile	Human transthyretin
Hennis, 2003^37^	Prospective cohort	General glaucoma	Barbados	3427	1	Statistical method: multiple regression analyses	Serum	HbA1c	HbA1c
Hysi, 2019^6^	Cross-sectional	General glaucoma	United Kingdom	113768 (Twins UK: 1763, UK Biobank: 103382, EPIC-Norfolk: 8623)^38–40^	2	Supervised: RF Statistical method: MR	Serum	Metabolic profile	O-methylascorbate (Vitamin C metabolite)
Iomdina, 2020^41^	Cross-sectional	POAG	Russia	76 (67 POAG, 9 deceased control donors)	1	Bioinformatics: GO (PANTHER), KEGG	Scleral biopsy	Scleral proteome	Vimentin, angiopoietin-related protein 7, annexin A2, serum amyloid P component, serum albumin, thrombospondin-4
Joachim, 2005^42^	Cross-sectional	POAG, NTG	Germany	66 (19 POAG, 17 NTG, 30 cataract controls)	1	Supervised: DA	Serum	Ocular antibody profile	Complex antibody patterns, especially retinal against antigens.
Joachim, 2007^43^	Cross-sectional	POAG, PXG	Germany	44 (15 POAG, 14 PXG, 30 cataract controls)	1	Supervised: DA	Aqueous humor	Ocular antibody profile	Heat shock protein 27, α-enolase, actin, GAPDH
Kouassi Nzoughet, 2020^26^	Cross-sectional	POAG	France	64 (34 POAG, 30 cataract controls)	2	Supervised: OPLS-DA, PLS-DA, RF, SVM, LASSO Unsupervised: PCA	Serum	Metabolic profile	Nicotinamide, hypoxanthine, xanthine, 1-methyl-6,7-dihydroxy-1,2,3,4-tetrahydroisoquinoline, N-acetyl-L-Leucine, arginine, RAC-glycerol 1-myristate, 1-oleoyl-RAC-glycerol, cystathionine
Lee, 2017^44^	Prospective cohort	NTG	Korea	71	1	Statistical method: univariate and multivariate regression analysis	Serum	Endothelin-1, macrophage chemoattractant protein-1 (MCP-1)	MCP-1
Li, 2020^45^	Phase 1: Cross-sectional, Phase 2: prospective cohort*	PACG	China	105	1	Statistical method: univariate and multivariate Cox proportional hazards regression analyses	Serum	Sex hormones, inflammatory cytokines	17-b-estradiol (E2), IL-6, IL-8, CRP, progesterone
Li, 2020^46^	Phase 1: Cross-sectional, Phase 2: prospective cohort*	PACG	China	94	1	Statistical method: logistic regression, Cox proportional hazards regression analyses	Serum	Oxidative stress markers (superoxide dismutase total antioxidant status, hydrogen peroxide, malondialdehyde, glutathione peroxidase, glutathione reductase)	Superoxide dismutase, total antioxidant status, malondialdehyde
Nusinovici, 2020^47^	Cross-sectional	POAG, PACG (DR, NPDR, AMD, PSC)	Singapore	10333 (Singapore Epidemiology of Eye Disease study)	1	Supervised: LASSO regression, GBM	Serum	Metabolic profile, HbA1c	Many significant biomarkers, not detailed.
Pan, 2020^48^	Cross-sectional	POAG	China	40 (16 POAG, 24 cataract controls)	3	Supervised: OPLS-DA Unsupervised: PCA Bioinformatics: KEGG	Aqueous humor	Metabolic profile	Glucose-1-phosphate, methylmalonic acid, N-cyclohexylformide 1, sorbitol, biotin, pelargonic acid, 2-mercaptoethanesulfonic acid 2, galactose 1, mannose 1, D-erythronolactone 2, dehydroascorbic acid 2, ribitol, D-talose
Myer, 2020^49^	Cross-sectional	PXG, POAG	USA	72 (31 PXG, 16 POAG, 25 non-glaucomatous controls)	3	Supervised: PLS-DA, SVM, ANN, deep learning Unsupervised: PCA Bioinformatics: KEGG	Aqueous humor	Metabolic profile	L-arginine, L-lysine, L-tyrosine, 2,4-diacetamido-2,4,6-trideoxy-beta-L-altrose, N(6)-acetonyllysine, 1-aminocyclropropane-1-carboxylate, L-histidine, C6H9N4O3P, C6H13NO6, 5-hydroxypentanoate, propylene glycol, creatinine, 2-hydroxy-butyrate, 3-methyl-2-oxovalerate, propylene glycol, 3-hydroxy isovalerate, pyruvate, choline
Qin, 2022^50^	Cross-sectional	PACG	China	521 (181 PACG, 340 non-PACG controls)		Supervised: PLS-DA Unsupervised: PCA Statistical method: Binary logistic regression	Serum	Plasma free fatty acids	C14:0, C16:1, C18:0, C20:0, C20:1, C:20:2, C:20:2, C:20:3, C:20:5, C22:1, C22:4, C22:5, C22:6, C24:0, Total x-3 FAs, Total x-6 FAs, x-3/x6, total FFAs, total SFAs, total PUFAs, total MUFAs
Sharma, 2018^51^	Cross-sectional	POAG	USA	47 (16 POAG, 32 cataract controls)	2	Bioinformatics: The Database for Annotation, Visualization and Integrated Discovery (DAVID), Ingenuity Pathway Analysis (IPA) Statistical method: multivariate logistic regression analysis	Aqueous humor	Proteomic profile	33 proteins.
Tang, 2021^52^	Cross-sectional	POAG	China	53 (28 POAG, 25 cataract controls)	3	Supervised: PLS-DA Bioinformatics: KEGG Statistical method: regression	Aqueous humor, serum	Metabolomic profile	Cyclic AMP, 2-methylbenzoic acid, 3′-sialyllactose, lysopc 18:0, dulcitol, lysopc 15:0, hypoxanthine Uric Acid, phenyllactate, xanthosine, lysopc 16:0, Lysopc 18:3, hydroxyphenyllactic acid, lysopa 16:0, lysopc 16:1, barbituric acid, L-3-phenyllactic acid, PAF C-16, N6-succinyl adenosine, hexadecanamide, lysopc 18:1, 3-(4-hydroxyphenyl)-propionic acid, N-lactoyl-phenylalanine, 9-hpode, D-mannitol, inosine, guanidinoethyl sulfonate, P-aminobenzoate, hydroxyacetone, 2-aminoadipic acid
de Voogd, 2006^53^	Prospective cohort	POAG, SOAG	Netherlands	3842	1	Statistical method: logistic regression	Serum	C-reactive protein (CRP)	None
Wang, 2019^54^	Cross-sectional	PSS	China	24 (12 PSS, 12 cataract controls)	4	Supervised: OPLS-DA Unsupervised: PCA Bioinformatics: KEGG Statistical method: logistic regression	Aqueous humor	Metabolic profile	3-Hydroxybutyric acid, allose, alpha-ketoisovaleric acid, aminoadipic acid, fumaric acid, glycine, homogentisic acid, ketoleucine, L-arabinose, L-glutamine, mannitol, phenylpyruvic acid, sorbitol, succinic acid. Pathways identified were alanine, aspartate, and glutamate metabolism, butanoate metabolism, citrate cycle, fructose and mannose metabolism, lysine degradation, nitrogen metabolism, phenylalanine metabolism, synthesis and degradation of ketone bodies, tyrosine metabolism, valine, leucine, and isoleucine biosynthesis and degradation.
Zhavoronkov, 2016^55^	Cross-sectional database	POAG	USA	35 (17 with POAG, 16 without POAG) from NCBI Gene Expression Omnibus^56^ and ArrayExpress^57^	2	Unsupervised: hierarchical clustering Bioinformatics: pathway analysis (Pathway Activation Strength)	Serum	Proteomic profile	TGFb, 50 other differentially activated signaling pathways
**Diagnostic Clarification**									
Barbosa breda, 2020^58^	Cross-sectional	POAG, NTG	Belgium, Portugal	90 (30 POAG, 30 NTG, 30 cataract controls)	2	Supervised: LDA, SVM Unsupervised: PCA	Aqueous humor*, serum	NMR of all AH metabolites	Alanine, N-acetylglutamate, lysine, glutamine, glutamate, valine, V-hydroxybutyrate, glutamine, a-ketogluterate, lysine, creatine, phosphocreatine, creatinine, a-ketogluterate, glucose, taurine, betaine, glucose, Ha of amino acids
Beutgen, 2019^27^	Cross-sectional	POAG	Germany	117, with age and gender matched controls with other eye diseases or healthy eyes Discovery phase - 12 (6 cases, 6 controls)** Validation phase - n = 105 (60 cases, 45 controls)**	1	Supervised: ANN - feed forward neural network (multilayer perceptron)	Serum	Serological antibody profile	CALD1, PGAM1, VDAC2, HSPD1, VIM
Burgess, 2015^59^	Cross-sectional	POAG	USA	144 (62 cases, 72 non-POAG controls)	2	Unsupervised: two-way hierarchical cluster analysis, Bioinformatics: pathway analysis (MetaboAnalyst), KEGG	Serum	Metabolomic profile	Significant pathways include galactose metabolism, fructose and mannose metabolism, steroid hormone biosynthesis
Igarashi, 2021^60^	Cross-sectional	POAG, SOAG, PXG	Japan	281 (193 with glaucoma, 88 without glaucoma)	1	Supervised: RF, SVM, LASSO regression	Aqueous humor	Autotaxin, and TGF-B levels	Autotaxin, TGF-β1, TGF-β3
Tokuda, 2012^61^	Cross-sectional	POAG	Japan	209 (115 POAG, 94 cataract controls)	1	Supervised: LDA, SVM, NBC, DT	Serum	Cytokine profile	3 cytokines: Fas Ligand, Eotaxin, MIG
**Treatment Decisions**									
Csosz, 2018^8^	Prospective cohort	POAG	Hungary	8	3	Unsupervised: hierarchical cluster analysis, pathway analysis Bioinformatics: GO Statistical method: linear regression	Tears	Proteomic profile	Elevated IL-6 and MMP1.
Liang, 2019^62^	Retrospective cohort	NVG	China	238	1	Statistical method: Cox proportional hazards regression analyses	Serum	HbA1c	HbA1c
Liu, 2021^63^	Cross-sectional	POAG	China	20 (10 case, 10 cataract control)	2	Bioinformatics: GO, KEGG Statistical method: linear regression	Aqueous humor	Proteomic profile	97 total proteins involved in glutathione metabolism (GSTP1), inflammation, immune responses, growth and development, cellular movement and vesicle-mediated transport.
Park, 2012^64^	Prospective cohort	POAG	Korea	36 (19 POAG, 17 cataract controls)	1	Statistical method: univariate and multivariate linear regressions	Aqueous humor, tenon tissue biopsy	VEGF	VEGF
Sakamoto, 2018^65^	Retrospective cohort	NVG	Japan	55	1	Statistical method: logistic regression	Serum	HbA1c, fasting blood glucose	Fasting blood glucose
Takayama, 2019^66^	Retrospective cohort	NVG	Japan	268	1	Statistical method: logistic regression	Serum	HbA1c	None (after regression).
Wakabayashi, 2012^67^	Retrospective cohort	NVG	Japan	52	1	Statistical method: univariate and multivariate logistic regression	Serum, aqueous humor, vitreous humour	HbA1c (serum), serum creatinine (serum), VEGF (vitreous, aqueous)	VEGF (vitreous)
Yildirim, 2008^68^	Prospective cohort	General glaucoma	Turkey	51 (34 DME, 17 healthy)	1	Statistical method: logistic regression	Serum	Matrix metalloproteinase-9 (MMP-9) and tissue inhibitor of MMP-2 (TIMP-2), HbA1c	TIMP-2 (after regression)
Zhang, 2018^69^	Prospective cohort	PACG	China	40	1	Statistical method: binary logistic regression, linear regression	Aqueous humor	Matricellular proteins	Secreted protein acidic and rich in cysteine
Zhu, 2019^9^	Case-control	PACG	China	104 (26 case, 78 control)	1	Statistical methods: univariate logistic regression	Aqueous humor	Thrombospondin-1 (TSP-1), TGF-B2	TSP-1, TGF-B2

* Relevant study phase

** In the discovery phase of the study the team sought to find relevant biomarkers, while in the validation phase the team tested AI models.

**Acronyms:** AI = artificial intelligence, AMD = age related macular degeneration, ANN = artificial neural network, DA = discriminant analysis, DME = diabetic macular edema, DR = diabetic retinopathy, DT = decision tree, EPIC-Norfolk = European Prospective Investigation into Cancer – Norfolk, GO = gene ontology, IOP = intraocular pressure, IPA = ingenuity pathways analysis, KEGG = Kyoto Encyclopedia of Genes and Genomes, LASSO = least absolute shrinkage and selection operator, NBC = Naive Bayes classifier, NPDR = nonproliferative diabetic retinopathy, NTG = normal tension glaucoma, NVG = neovascular glaucoma, OPLS-DA = orthogonal partial least-squares discriminant analysis, PACG = primary angle closure glaucoma, PCA = principal component analysis, PDR = proliferative diabetic retinopathy, PLS-DA = partial least-squares discriminant analysis, POAG = primary open angle glaucoma, PSS = Posner-Schlossman Syndrome, PXG = pseudoexfoliation glaucoma, RF = random forest, SOAG = secondary open angle glaucoma, SVM = support vector machine

### Biofluid markers

Serum made up 54.5% of the biofluids analyzed, aqueous humour was 36.3%, tissue biopsy was 4.5%, vitreous humour was 2.3%, and tears were 2.3%. While many studies looked at entire metabolomic or proteomic profiles to determine changes in POAG, there was heterogeneity in the data with reporting of over 175 unique, differentially expressed biomarkers, over 75 biological pathways implicated in POAG development, and over 50 unique biomarkers determined to be non-significant.^8,9,25,27,28,33,34,36,41–49,51–53,55,58–61,63,64^ Studies each identified between one and 773 differentially expressed biomarkers. Many studies using unsupervised AI to look for differentially expressed markers did not report all non-significant biomarkers investigated. The only biomarkers implicated in POAG development by multiple studies were glycemic level, TGF-β1, alanine, glutamine, leucine, taurine, hypoxanthine, and sorbitol.^25,34,47,55,58,60^ Glutamine was the most commonly implicated, referenced in three studies.^34,47,58^ Pathways implicated in POAG by multiple studies included the glycolytic pathway, inflammation, autoimmune mechanisms, extracellular matrix-receptor interaction, cellular transport, cell-cell signalling, and signal transduction.^27,28,41,42,51,63^ The glycolytic, inflammatory, and autoimmune pathways were the most commonly implicated, each referenced by three studies.^27,28,41,42,51^ Despite the lack of similar findings between studies, various diagnostic and prognostic predictive AI models were developed using identified biomarkers. However, biomarker selection variably affected the accuracy of these AI algorithms.^48,51^

In the examination of NVG development, particularly in a post-operative context, HbA1c was found to be a predictive biomarker, as was vascular endothelial growth (VEGF).^62,64,66,67^ However, four studies conflicted with these findings, determining both HbA1c and VEGF to be insignificant to NVG development.^35,65–67^ While 15 biomarkers and one pathway were found to be differentially expressed in NTG over three studies, there was no overlapping findings between studies (Table 1).^42,44,58^ Similarly more than 20 biomarkers were identified in pseudoexfoliation glaucoma (PXG), more than 20 biomarkers were identified in PACG, and more than 10 biomarkers were identified in Posner-Schlossman Syndrome (PSS), but none were confirmed by more than one study (Table 1).

### Applications of AI and bioinformatics

A total of 24 studies used a singular type of AI in their analysis, with 14 using a statistical method (regression), eight using supervised AI, and two using bioinformatics alone (Table 1). Fourteen studies used two or more types of AI in their analysis. ANN, discriminant analysis, support vector machine, random forest (RF), deep learning, and Naive Bayes Classifier were among the supervised tools utilized to develop models that differentiated between glaucoma subtypes and controls, with accuracy reported in 13 papers.^25–27,34,36,48,49,51,52,54,58,60,61^ Nine of the papers reporting differentiating accuracy has the objective of identifying disease characteristics, while four were intended to develop diagnostic tools. While the goals of predictive models were variable between studies, all models sought to autonomously identify glaucoma patients from controls or other glaucoma subtypes given a test set of labelled samples, with the identifying disease characteristics focusing on the identification of a characteristic biomarker and the diagnostic studies focusing on maximization of algorithm accuracy (Supplemental Table 1). For example, Tang et al. (2021) collected aqueous humour samples from POAG patients undergoing various glaucoma surgeries and from controls undergoing cataract extraction surgery. Partial Least-Squares Discriminant Analysis (PLS-DA) was applied to determine the differentially expressed metabolites between the two groups following univariate analysis of the clinical data.^52^ The PLS-DA in this case identified metabolites in a complex dataset more accurately and efficiently than would have been possible using traditional statistical methods.^52^ PLS-DA and other algorithms, such as RF analysis, were then used to determine the diagnostic power of the discovered biomarkers. Finally, KEGG was applied to the findings to identify the altered underlying physiological processes and potential therapeutic strategies.^52^ In another instance, Anton Apreutesei et al. (2018) applied ANNs to accurately predict predicting ocular changes associated with diabetes in glaucoma patients; these ANNs allowed for the modelling of complex nonlinear relationships, contributions from large numbers of predictor variables, and high predictive accuracy, tasks that traditional statistical tools cannot achieve.^25^

In studies that reported accuracy as the percentage of cases that were accurately classified, predictive accuracy ranged from 51% to 95%; generally ANNs were the most accurate while support vector machine were the least accurate.^26,49,61^ The accuracies of all studies are summarized in Table 2. Selection of the AI algorithm had considerable influence on model accuracy. For example, Tokuda et al. (2012) compared linear discriminant analysis, support vector machine, Naive Bayes Classifier, and decision tree models using the same biomarkers, calculating the lowest accuracy at 51.2% (polynomial support vector machine) and the highest at 74.4% (Naive Bayes Classifier).^61^ Accuracy was also measured as sensitivity and specificity, with sensitivity ranging from 81–90% and specificity ranging from 87–93%, indicating excellent accuracy.^27,36^ The accuracy of tools was most commonly reported as area under receiver operating curve (AUROC), a graphical description of sensitivity and specificity; reported AUROCs ranged from 0.58–0.93, with the majority being >0.85.^9,25,26,34,48,52,58,60,70^ Sharma *et al.* developed a predictive model using bioinformatics in Database for Annotation, Visualization and Integrated Discovery (DAVID) and Ingenuity Pathway Analysis (IPA) and regression analysis, demonstrating AUROCs >0.75.^51^ However, this model used previously identified risk factors rather than a complete biofluid profile. In studies that compared predictive models that used different biomarkers, biomarker selection had significant effect on model accuracy; for example, Pan *et al.* (2020) had an AUROC of 0.62 using d-erythronalactone 2, but demonstrated an AUROC of 0.86 with Galactose 1.^48^

**Table 2.** Predictive accuracy of AI algorithms.

First Author, Publication year	AI algorithm	Accuracy	Description
Anton Apreutesei, 2018^25^	ANN (MLP–multilayer perceptron, Jordan Elman Network)	AUROC = 0.420 (HbA1c), 0.439 (IOP), 0.700 (C/D), 0.770 (MD)	Prediction of diabetic eye disease using biomarkers and clinical characteristics.
Barbosa Breda, 2020^58^	ANN	AUROC = 0.91 (LDA), 0.93 (SVM)	Differentiated glaucoma from health controls. The model was unable to differentiate between NTG and POAG (<0.65 AUROC).
Beutgen, 2019^33^	LDA, SVM, PCA	Sensitivity = 81%, specificity = 93% AUROC = 0.875	Classify patients as POAG or control.
Buisset, 2019^34^	PCA, PLS-DA, regression	AUROC = 0.89 (PLS-DA with test sets with p-values of 0.0087)	Classify patients as POAG or control.
Grus, 2008^36^	DA, ANN	Sensitivity = 90%, specificity = 87%	Classify patients as POAG or control.
Igarashi, 2021^60^	RF, SVM, LASSO	AUC (LASSO using ATX, TGF-B1, TFG-B2, TGF-B3) = 0.675 (POAG vs control), 0.729 (SOAG vs control), 0.966 (PXG vs control), 0.670 (POAG vs SOAG), 0.913 (POAG vs PXG), 0.834 (SOAG vs PXG) AUC (LASSO using ATX, TGF-B3) = 0.607 (POAG vs control), 0.747 (SOAG vs control), 0.967 (PXG vs control), 0.694 (POAG vs SOAG), 0.860 (POAG vs PXG), 0.854 (SOAG vs PXG)	Differentiate between glaucoma subtypes (POAG, SOAG, PXG) and controls.
Myer, 2020^49^	PCA, PLS-DA, SVM, DL, ANN, KEGG	Enhanced ANN accuracy = >90% Deep learning accuracy = >80%) SVM accuracy was variable and unpredictable	Differentiate between POAG, PXG, and controls.
Kouassi Nzoughet, 2020^26^	PCA, OPLS-DA, PLS-DA, RF, SVM, LASSO	LASSO generated 100 models, with 75% having AUC of >0.8 (median AUC = 0.86, mean AUC = 0.84). 303 models were used in variable selection as they exhibited very good predictive performances on the test set (AUC ≥ 0.9). Accuracy (eight metabolite panel) = 93.01% (control prediction), 82.43% glaucoma prediction) Accuracy (PLS-DA using nicotinamide and N-acetyl L-leucine) = 73.7% Accuracy (SVM using nicotinamide and N-acetyl L-leucine) = 71.1%	Classify patients as POAG or control.
Pan, 2020^48^	PCA, OPLS-DA, KEGG	AUC = 0.62–0.85 (dependant on metabolite used in model)	Classify patients as POAG or control.
Sharma, 2018^51^	DAVID, IPA, regression	AUC = 0.732–0.793 (dependant on metabolite used in model)	Classify patients as POAG or control.
Tang, 2021^52^	PLS-DA, KEGG, regression	AUC = 0.57–0.87 (dependant on metabolite used in model), with the strongest biomarkers having AUCs of 0.87 (AMP), 0.75 (2-methylbenzoic acid), 0.73 (3′-sialyllactose), 0.76 (N-lac-phe)	Classify patients as POAG or control.
Tokuda, 2012^61^	LDA, SVM, NBC, DT	LDA (integrated model with sampling): accuracy = 65.5%, sensitivity = 61.1%, specificity = 71.7% Linear SVM (integrated model with sampling): accuracy = 66.8%, sensitivity = 64.0%, specificity = 70.6% Polynomial SVM (integrated model with sampling): accuracy = 62.4%, sensitivity = 48.0%, specificity = 82.7% RBF SVM (integrated model with sampling): accuracy = 74.0%, sensitivity = 80.5%, specificity = 65.0% NBC (integrated model with sampling): accuracy = 69.8%, sensitivity = 64.4%, specificity = 77.5% DT (integrated model with sampling): accuracy = 61.7%, sensitivity = 66.8%, specificity = 54.5% Note that genotype and cytokine models are also reported, as are accuracies using single analysis.	Classify patients as POAG or control.
Wang, 2019^54^	PCA, OPLS-DA, KEGG, regression	AUC = 0.70833–0.88889 (dependant on metabolite used in model)	Classify patients as PSS or control.

**Acronyms**: AI = artificial intelligence, AUC = area under curve, AUROC = area under receiver operating curve, ANN = artificial neural network, DA = discriminant analysis, DT = decision tree, IOP = intraocular pressure, IPA = ingenuity pathways analysis, KEGG = Kyoto Encyclopedia of Genes and Genomes, LASSO = least absolute shrinkage and selection operator, NBC = Naive Bayes classifier, OPLS-DA = orthogonal partial least-squares discriminant analysis, PACG = primary angle closure glaucoma, PCA = principal component analysis, PDR = proliferative diabetic retinopathy, PLS-DA = partial least-squares discriminant analysis, POAG = primary open angle glaucoma, PSS = Posner-Schlossman Syndrome, PXG = pseudoexfoliation glaucoma, RF = random forest, SOAG = secondary open angle glaucoma, SVM = support vector machine.

Findings from unsupervised AI analyses were used to either select a differentiating biomarker for use in subsequent diagnostic algorithms or to explain glaucoma pathogenesis.^8,26,28,34,48–50,55,58,59,71^ Ten studies used unsupervised AI in conjunction with other analytical methods.^8,26,28,34,48–50,55,58,59,71^ Bioinformatics tools were commonly used in conjunction with unsupervised analysis, providing interpretation of the results and linking differentiating biomarkers to biological pathways, genes, or specific metabolic processes.^8,28,33,48,49,55,62^ This enabled consideration of potential therapeutic targets, or provided direction for future study of pathogenic mechanism.^8,26,28,33,48,49,52,55,62^

AI statistical methods, namely regression analysis, were the most commonly employed class of analytic techniques. A total of 14 papers used regression models exclusively to identify independent factors related to an outcome of interest.^9,35,37,44–46,53,62,64–69^ As regression analysis is less useful in the study of highly dimensional data, studies using regression alone focused on a smaller number of biofluid markers and clinical characteristics as opposed to the entire metabolic, proteomic, or lipidomic profile as seen in the supervised and unsupervised AI analyses. Regression was typically used to determine longitudinal association between a biomarker and a clinical outcome or condition with the intention of identifying risk factors or an intervention to prevent an outcome or disease progression.^9,35,37,44–46,53,62,64–69^ None of these studies used test/validation sets in their analysis.

### Quality appraisal

The included studies were generally of high quality, with 18 having moderate risk of bias, 16 having low risk of bias, and five having high risk of bias (Supplemental Materials 3). Risk of bias was evenly distributed between study characterizations; in the five studies with high risk of bias, three were characterized as Identifying Disease Characteristics, with one of both Treatment Decisions and Diagnostic Clarification studies having high risk of bias. Given the exploratory nature of many included studies, non-significant findings were often omitted, introducing reporting bias. Cross-sectional studies had inconsistent reporting of participant inclusion criteria and often failed to provide robust descriptions of exposure measurement protocols (45%). In contrast, cohort studies explained participant inclusion protocols and exposure criteria in-depth, but many did not describe loss to follow-up (71%) and none described strategies to mitigate incomplete follow-up (100%). All studies described their biomarker measurement protocols (assays, laboratory parameters) in detail, but used very small volumes of biofluid, potentially introducing measurement error. Importantly, very few of the studies using complex AI (supervised, unsupervised, bioinformatics) explained the rational for AI selection, or the algorithms activities; black-box models reduce the reproducibility of the study and compromise the external validity.

## Discussion

This systematic review describes the current evidence available for AI and bioinformatic analysis of biofluid markers in glaucoma. AI algorithms using biofluid markers were unable to provide a definitive characteristic biomarker, but predictive tools using these markers demonstrated strong preliminary results. While predictive models using AI have not been tested in a clinical context, AI tools such as discriminant analysis and artificial neural network displayed strong differentiating ability between glaucoma patients and controls, with average sensitivity and specificity of >85%. However, there is notable variation in the differentially expressed biomarkers found to be significant between studies; >300 biomarkers and pathways were reported to be significantly different in glaucoma patients, with little confirmation between studies. Given this heterogeneity, no clear characteristic biomarker that could provide insight into glaucoma pathogenesis or aid in diagnosis has yet emerged using AI analysis of biofluid markers.

With studies using as few as one biomarker in their analysis, and others using highly dimensional data detailing as many as 773 differentially expressed proteins, some heterogeneity in biomarker reporting was expected. However, the majority of biomarkers were exclusively reported on by one study, with most utilized marker for POAG, glutamate, implicated by only three papers.^34,47,58^ Only nine total biomarkers were noted to be significantly different in glaucoma patients across two or more studies.^8,9,25,27,28,34,36,41,42,44–49,51–53,55,58–61,63,64^ Further, as there was minimal reporting of non-significant biomarkers, it is likely that the variability in findings is even greater than reported in the literature. With no strong, replicable, characteristic biomarkers for each glaucoma subtype, the biomarkers provide little insight in glaucoma etiology. As such, the utility of biofluid markers as a tool to guide therapeutic development is limited.

The underlying causes of biomarker variation are challenging to identify due to the variable study design and the paucity of detail of the AI models described by the included studies. For one, while they often sought to identify biomarkers unique to a glaucoma subtype, each studied addressed different research questions. One of the important considerations is the small quantities of biofluid analyzed, particularly in the study of ocular fluid such as vitreous or aqueous humour where aliquots range from 200 μL to as low as 20 μL (with majority of studies having approximately 100 μL).^9,28,34,36,48,49,51,52,54,58,60,63,64,67,69^ While these volumes may be analyzed using commercial assays, such small quantities are more susceptible to changes in the microenvironment, an issue further exacerbated by differing sample dilutions, storage, and handling techniques. Also notable was differing measurement tools, such as the analysis of cytokines using ELISA or other various commercial assays. Additionally, many of the cross-sectional AI studies did not provide robust explanation of their study population, leaving potentially confounding experimental conditions or patient characteristics such as medication use, comorbidities, or ethnicity that could alter biomarker concentrations, particularly in studies with smaller sample sizes.^28,36,41,48,49,51,52,54,55^ It is important to note that a large proportion of the studies using complex AI in their analyses take a “black-box” approach and provide sparse rational about algorithm selection and do not detail how the algorithm structure interacts with the data to extract patterns, meaning each study could be selecting biomarkers using vastly different parameters or using different data structures.^72^ Finally, AI algorithms have distinct applications, strengths, and weaknesses. As such, selection of AI tools is crucial for suitable and accurate use. While no instances of inappropriate algorithm selection were noted in the included studies, researchers and clinicians should seek to carefully understand AI tools before using them.

Despite the limited value of the biomarkers in understanding disease pathogenesis, AI may offer strong predictive and diagnostic value for patients with glaucoma. Supervised AI was particularly valuable in glaucoma diagnostics, where AI tools were able to classify glaucoma cases from controls and detect patterns that were previously unidentified or too complex to be interpreted by other analytical tools.^25^ AI models were able to separate glaucoma patients from controls with accuracy as high as 95%, with most demonstrating AUROCs of >85%. These models could be comparable to, and in some cases better than, human diagnosis of glaucoma using imaging, which a study by Yang *et al.* (2019) found to be 90.0% and 94.8% accuracy for two independent diagnosticians.^73^ While selection of AI algorithm had significant effect on accuracy, there was no clear advantage in precision for models that used multiple types of AI (e.g., supervised with unsupervised).^36,54,58,60^ There was also no clear trends in accuracy of models derived from different biofluids.

Glaucoma diagnosis using imaging-based AI tools have been reported to have higher accuracy than the studies included in this review, although biofluid AI models are earlier in development.^19,20,24^ As such, biofluid marker AI models may be utilized for glaucoma diagnosis to augment existing imaging-based tools to increase accuracy. Tokuda *et al.* (2012) demonstrate this principle, showing increased glaucoma diagnostic accuracy with combined genotype and cytokine data, when compared to each independantly.^61^ As frequent procedures are common in the glaucoma patient population, serum or ocular fluids such as aqueous humour may be readily available for use in clinical tools. An important limitation of the AI tools described in the included papers is the lack of demonstrated results in a clinical context; all of the included studies and most other studies identified in the literature are purely investigational and have not been tested or deployed with real patients. Additionally, expertise is required to implement AI tools and interpret AI findings, a skillset that might not be common amongst clinicians and for which there is limited access to training opportunities. Finally, biomarker extraction and analysis can be labour intensive and require specialized tools, presenting a financial barrier.

As AI tools progress towards use in clinical practice, it is important that the medical community engage in active preparation to prevent misuse and poor technical understanding.^74^ These issues arise not from the efficacy of the technology, but rather it's thoughtful implementation in clinical settings.^74^ None of the preliminary research of AI analysis of biofluid markers were directly used to inform clinical decision making or were applied in a clinical context, where it is crucial to examine integration into clinical workflow and establish efficacy prior to use by clinicians. Future efforts should seek to validate AI models in different patient populations to support clinical tool development, which is of particular importance as many studies did not describe the study population in detail. For example, a comparison of diagnostic accuracy between an AI algorithm and a human grader using the gold-standard diagnostic technique in various patient populations could provide insight into the diagnostic utility of AI analysis of biofluid markers. Additionally, future studies using AI analysis of biofluid markers should ensure complete description of both their analytical methods, study population, and loss to follow-up.

## Conclusion

In this review we present studies that use AI or bioinformatics to analyze biofluid markers in glaucoma. The use of models such as discriminant analysis and artificial neural network were able to distinguish glaucoma patients from controls with high sensitivity and specificity. These tools could be used to augment existing clinical tools and inform clinical decision making with the growing burden of glaucoma.^1,2^ While the insight AI analysis provided into differentially expressed biomarkers is valuable in pathogenic exploration, no clear pathogenic mechanism in glaucoma has yet emerged. Future studies should seek to validate AI models with the goal of clinical tool development.

## Supplemental Material

sj-docx-1-ejo-10.1177_11206721221140948 - Supplemental material for The role of artificial intelligence in analysis of biofluid markers for diagnosis and management of glaucoma: A systematic review Click here for additional data file. Supplemental material, sj-docx-1-ejo-10.1177_11206721221140948 for The role of artificial intelligence in analysis of biofluid markers for diagnosis and management of glaucoma: A systematic review by Aidan Pucchio, Saffire Krance, Daiana R Pur, Arshpreet Bassi, Rafael Miranda and Tina Felfeli in European Journal of Ophthalmology

sj-docx-2-ejo-10.1177_11206721221140948 - Supplemental material for The role of artificial intelligence in analysis of biofluid markers for diagnosis and management of glaucoma: A systematic review Click here for additional data file. Supplemental material, sj-docx-2-ejo-10.1177_11206721221140948 for The role of artificial intelligence in analysis of biofluid markers for diagnosis and management of glaucoma: A systematic review by Aidan Pucchio, Saffire Krance, Daiana R Pur, Arshpreet Bassi, Rafael Miranda and Tina Felfeli in European Journal of Ophthalmology

sj-docx-3-ejo-10.1177_11206721221140948 - Supplemental material for The role of artificial intelligence in analysis of biofluid markers for diagnosis and management of glaucoma: A systematic review Click here for additional data file. Supplemental material, sj-docx-3-ejo-10.1177_11206721221140948 for The role of artificial intelligence in analysis of biofluid markers for diagnosis and management of glaucoma: A systematic review by Aidan Pucchio, Saffire Krance, Daiana R Pur, Arshpreet Bassi, Rafael Miranda and Tina Felfeli in European Journal of Ophthalmology
