# Supplementary material for: The role of artificial intelligence in analysis of biofluid markers for diagnosis and management of glaucoma: A systematic review
Source: Eur J Ophthalmol. 2022 Nov 25;33(5):1816–33. doi: 10.1177/11206721221140948 (PMC10469503; doi:10.1177/11206721221140948)
Supplement: sj-docx-1-ejo-10.1177_11206721221140948 - Supplemental material for The role of artificial intelligence in analysis of biofluid markers for diagnosis and management of glaucoma: A systematic review [file sj-docx-1-ejo-10.1177_11206721221140948.docx]

**Supplemental Materials 1.** Search strategy utilized for five electronic databases (EMBASE, Medline, Cochrane Central Register of Controlled Trials, Cochrane Database of Systematic Reviews, Web of Science)

**Embase**

1. (ophth* or ocular or intraocular or eye* or retina* or macula* or fovea* or uvea* or sclera* or cornea* or conjunctiva* or iris or "vitreous body" or "vitreous humo?r" or "vitreous fluid" or vitreo* or "aqueous humo?r" or "aqueous fluid" or tears or ((tear or lacrimal) adj fluid) or glaucoma or retinop* or retinoblastoma or uveitis or iritis or choroiditis or retinitis or chorioretinitis or conjunctivitis or endophthalmitis or cataract* or ?????????opia or "optic atrophy" or "optic neuropathy" or vitrectomy or phacoemulsification or trabeculotomy or (paracentesis adj3 "anterior chamber")).tw.
2. ("precision medicine" or "precision health" or "personalized medicine" or "personalized proteomics" or thera?nostic? or "tailored medicine" or "artificial intelligence" or "machine learning" or "deep learning" or algorithm? or ((supervised or unsupervised or biased or unbiased or bayesian or hierarchical or neur??al) adj (cluster* or learning or learner? or classifi* or network?)) or "k-nearest neighbo?r?" or "naive bayes" or (decision adj (tree? or forest? or jungle?)) or "random forest?" or "gradient-boost*" or "support vector machine" or "k-means" or "association rules" or "recursive partitioning" or "discriminant analysis" or "feature selection" or ((linear or nonlinear or "non-linear" or logistic or ordinal or poisson or quantile or analysis) adj1 (regression? or model?)) or bioinformatic? or "gene ontology" or "Kyoto Encyclopedia of Genes and Genomes" or "KEGG" or ((progress* or regress* or recover* or respond* or response*) and (predict* or stratif*))).tw.
3. (Proteomic? or proteome? or metabolomic? or metabolome? or lipidomic? or lipidome? or "?????inflammatory protein?" or "?????inflammatory marker" or cytokine? or interleukin? or lymphokine? or monokine? or interferon? or "colony stimulating factor?" or chemokine? or "growth factor?" or "necrosis factor?" or "chemotactic protein?" or "adhesion molecule?" or "adhesion protein?" or "matrix metalloproteinase-2" or myeloperoxidase? or "tissue inhibitor of metalloproteinase-2" or "macrophage inflammatory protein-1" or "brain-derived neurotrophic factor" or angiopoietin? or ((hemoglobin or haemoglobin) adj1 (a1c or glycated)) or hba1c or "c reactive protein" or "c-reactive protein" or crp or hscrp or "hs-crp" or ((protein or biomarker) and (concentration? or level? or quantif* or quantit* or mass spectrometry or iTRAQ or MALDI or SELDI or assay))).tw.
4. ophthalmology/
5. eye/ or anterior eye chamber/ or anterior eye segment/ or aqueous humor/ or exp conjunctiva/ or exp cornea/ or eye fundus/ or eyeball/ or exp lens/ or ocular blood vessel/ or ophthalmic artery/ or optic disk/ or palpebral fissure/ or posterior eye chamber/ or posterior eye segment/ or exp retina/ or exp sclera/ or sphincter pupillae muscle/ or tenon capsule/ or trabecular meshwork/ or exp uvea/ or vitreous body/
6. lacrimal fluid/
7. eye disease/ or exp accommodation disorder/ or exp conjunctiva disease/ or exp cornea disease/ or exp dry eye/ or exp eye burning/ or exp eye chamber disease/ or exp eye discharge/ or exp eye discomfort/ or exp eye edema/ or exp eye infection/ or exp eye inflammation/ or exp eye injury/ or exp eye irritation/ or exp eye jaundice/ or exp eye malformation/ or exp eye pain/ or exp eye redness/ or exp eye swelling/ or exp eye toxicity/ or exp eye tumor/ or exp glaucoma/ or exp intraocular hemorrhage/ or exp intraocular pressure abnormality/ or exp lens disease/ or exp ocular albinism/ or exp ocular fibrosis/ or exp ocular pruritus/ or exp ocular surface disease/ or exp optic nerve disease/ or exp photophobia/ or exp pupil disease/ or exp retina disease/ or exp sclera disease/ or exp uvea disease/ or exp visual disorder/ or exp vitreous disease/
8. exp vitrectomy/
9. exp phacoemulsification/
10. exp trabeculectomy/
11. personalized medicine/
12. theranostic nanomedicine/
13. algorithm/
14. exp clustering algorithm/
15. artificial intelligence/
16. exp machine learning/
17. "decision tree"/
18. bioinformatics/
19. exp regression analysis/
20. discriminant analysis/
21. gene ontology/
22. proteomics/ or comparative proteomics/ or immunoproteomics/ or exp pharmacoproteomics/ or phosphoproteomics/ or proteogenomics/ or secretomics/
23. proteome/
24. metabolomics/
25. metabolome/
26. lipidomics/
27. lipidome/
28. exp cytokine/
29. exp cell adhesion molecule/
30. myeloperoxidase/
31. "tissue inhibitor of metalloproteinase 1"/
32. "tissue inhibitor of metalloproteinase 2"/
33. brain derived neurotrophic factor/
34. exp angiopoietin/
35. exp "peptides and proteins"/ec [Endogenous Compound]
36. biological marker/ec [Endogenous Compound]
37. 1 or 4 or 5 or 6 or 7 or 8 or 9 or 10
38. 2 or 11 or 12 or 13 or 14 or 15 or 16 or 17 or 18 or 19 or 20 or 21
39. (concentration? or level? or quantif* or quantit* or mass spectrometry or iTRAQ or MALDI or SELDI or assay).tw.
40. (35 or 36) and 39
41. 3 or 22 or 23 or 24 or 25 or 26 or 27 or 28 or 29 or 30 or 31 or 32 or 33 or 34 or 40
42. 37 and 38 and 41
43. limit 42 to conference abstracts
44. limit 42 to animal studies
45. limit 44 to human
46. limit 42 to "review"
47. 44 not 45
48. 42 not (43 or 46 or 47)

**Medline**

1. (ophth* or ocular or intraocular or eye* or retina* or macula* or fovea* or uvea* or sclera* or cornea* or conjunctiva* or iris or "vitreous body" or "vitreous humo?r" or "vitreous fluid" or vitreo* or "aqueous humo?r" or "aqueous fluid" or tears or ((tear or lacrimal) adj fluid) or glaucoma or retinop* or retinoblastoma or uveitis or iritis or choroiditis or retinitis or chorioretinitis or conjunctivitis or endophthalmitis or cataract* or ?????????opia or "optic atrophy" or "optic neuropathy" or vitrectomy or phacoemulsification or trabeculotomy or (paracentesis adj3 "anterior chamber")).tw.
2. ("precision medicine" or "precision health" or "personalized medicine" or "personalized proteomics" or thera?nostic? or "tailored medicine" or "artificial intelligence" or "machine learning" or "deep learning" or algorithm? or ((supervised or unsupervised or biased or unbiased or bayesian or hierarchical or neur??al) adj (cluster* or learning or learner? or classifi* or network?)) or "k-nearest neighbo?r?" or "naive bayes" or (decision adj (tree? or forest? or jungle?)) or "random forest?" or "gradient-boost*" or "support vector machine" or "k-means" or "association rules" or "recursive partitioning" or "discriminant analysis" or "feature selection" or ((linear or nonlinear or "non-linear" or logistic or ordinal or poisson or quantile or analysis) adj1 (regression? or model?)) or bioinformatic? or "gene ontology" or "Kyoto Encyclopedia of Genes and Genomes" or "KEGG" or ((progress* or regress* or recover* or respond* or response*) and (predict* or stratif*))).tw.
3. (Proteomic? or proteome? or metabolomic? or metabolome? or lipidomic? or lipidome? or "?????inflammatory protein?" or "?????inflammatory marker" or cytokine? or interleukin? or lymphokine? or monokine? or interferon? or "colony stimulating factor?" or chemokine? or "growth factor?" or "necrosis factor?" or "chemotactic protein?" or "adhesion molecule?" or "adhesion protein?" or "matrix metalloproteinase-2" or myeloperoxidase? or "tissue inhibitor of metalloproteinase-2" or "macrophage inflammatory protein-1" or "brain-derived neurotrophic factor" or angiopoietin? or ((hemoglobin or haemoglobin) adj1 (a1c or glycated)) or hba1c or "c reactive protein" or "c-reactive protein" or crp or hscrp or "hs-crp" or ((protein or biomarker) and (concentration? or level? or quantif* or quantit* or mass spectrometry or iTRAQ or MALDI or SELDI or assay))).tw.
4. exp Ophthalmology/cl, di, dg, ec, pd, px, sn, sd, su, th, td, ed, es, hi, is, mt, og, rt, st [Classification, Diagnosis, Diagnostic Imaging, Economics, Pharmacology, Psychology, Statistics & Numerical Data, Supply & Distribution, Surgery, Therapy, Trends, Education, Ethics, History, Instrumentation, Methods, Organization & Administration, Radiotherapy, Standards]
5. eye/ or exp anterior eye segment/ or "anterior capsule of the lens"/ or conjunctiva/ or meibomian glands/ or exp "pigment epithelium of eye"/ or exp posterior eye segment/ or exp retina/ or sclera/ or tenon capsule/ or exp uvea/
6. Tears/
7. eye diseases/ or cogan syndrome/ or exp conjunctival diseases/ or exp corneal diseases/ or exp eye abnormalities/ or exp eye diseases, hereditary/ or exp eye hemorrhage/ or exp eye infections/ or exp eye injuries/ or exp eye manifestations/ or exp eye neoplasms/ or exp lens diseases/ or exp ocular hypertension/ or ocular hypotension/ or exp optic nerve diseases/ or exp pupil disorders/ or exp refractive errors/ or exp retinal diseases/ or exp scleral diseases/ or exp uveal diseases/ or exp vision disorders/ or vitreous detachment/
8. Vitrectomy/ae, ec, ed, es, hi, is, mt, mo, nu, px, rh, st, sn, td [Adverse Effects, Economics, Education, Ethics, History, Instrumentation, Methods, Mortality, Nursing, Psychology, Rehabilitation, Standards, Statistics & Numerical Data, Trends]
9. Phacoemulsification/ae, cl, ec, ed, hi, is, mt, mo, nu, px, rh, st, sn, td [Adverse Effects, Classification, Economics, Education, History, Instrumentation, Methods, Mortality, Nursing, Psychology, Rehabilitation, Standards, Statistics & Numerical Data, Trends]
10. Trabeculectomy/nu, px, rh, st, sn, td, ae, cl, ec, ed, hi, is, mt, mo [Nursing, Psychology, Rehabilitation, Standards, Statistics & Numerical Data, Trends, Adverse Effects, Classification, Economics, Education, History, Instrumentation, Methods, Mortality]
11. Precision Medicine/ae, cl, ec, es, hi, is, mt, mo, nu, px, st, sn, td [Adverse Effects, Classification, Economics, Ethics, History, Instrumentation, Methods, Mortality, Nursing, Psychology, Standards, Statistics & Numerical Data, Trends]
12. Theranostic Nanomedicine/
13. exp algorithms/
14. Neural Networks, Computer/
15. Decision Trees/
16. exp Regression Analysis/
17. Discriminant Analysis/
18. exp Proteomics/cl, ec, ed, es, hi, is, mt, og, st, sn, td [Classification, Economics, Education, Ethics, History, Instrumentation, Methods, Organization & Administration, Standards, Statistics & Numerical Data, Trends]
19. Proteome/
20. exp Metabolomics/cl, ec, ed, es, hi, is, mt, og, st, sn, td [Classification, Economics, Education, Ethics, History, Instrumentation, Methods, Organization & Administration, Standards, Statistics & Numerical Data, Trends]
21. Metabolome/
22. exp Cytokines/
23. exp Cell Adhesion Molecules/
24. Matrix Metalloproteinase 2/
25. Peroxidase/
26. "Tissue Inhibitor of Metalloproteinase-1"/
27. "Tissue Inhibitor of Metalloproteinase-2"/Brain-Derived Neurotrophic Factor/
28. exp Angiopoietins/
29. Gene Ontology/
30. exp Proteins/
31. exp Peptides/
32. Biomarkers/
33. (concentration? or level? or quantif* or quantit* or mass spectrometry or iTRAQ or MALDI or SELDI or assay).tw.
34. (31 or 32 or 33) and 3
35. 1 or 4 or 5 or 6 or 7 or 8 or 9 or 10
36. 2 or 11 or 12 or 13 or 14 or 15 or 16 or 17 or 30
37. 3 or 18 or 19 or 20 or 21 or 22 or 23 or 24 or 25 or 26 or 27 or 28 or 29 or 35
38. 36 and 37 and 38
39. limit 39 to animals
40. limit 40 to humans
41. 40 not 41
42. limit 39 to "review articles"
43. 39 not (42 or 43)

**Web of Science**

1. (TI=(ophth* or ocular or intraocular or eye* or retina* or macula* or fovea* or uvea* or sclera* or cornea* or conjunctiva* or iris or "vitreous body" or "vitreous humo$r" or "vitreous fluid" or vitreo* or "aqueous humo$r" or "aqueous fluid" or tears or ((tear or lacrimal) NEAR/1 fluid) or glaucoma or retinop* or retinoblastoma or uveitis or iritis or choroiditis or retinitis or chorioretinitis or conjunctivitis or endophthalmitis or cataract* or *opia or "optic atrophy" or "optic neuropathy" or vitrectomy or phacoemulsification or trabeculotomy.tw. or (paracentesis NEAR/3 "anterior chamber")) or AB=(ophth* or ocular or intraocular or eye* or retina* or macula* or fovea* or uvea* or sclera* or cornea* or conjunctiva* or iris.tw. or "vitreous body" or "vitreous humo$r" or "vitreous fluid" or vitreo* or "aqueous humo$r" or "aqueous fluid" or tears or ((tear or lacrimal) NEAR/1 fluid) or glaucoma or retinop* or retinoblastoma or uveitis or iritis or choroiditis or retinitis or chorioretinitis or conjunctivitis or endophthalmitis or cataract* or "optic atrophy" or "optic neuropathy" or vitrectomy or phacoemulsification or trabeculotomy or (paracentesis NEAR/3 "anterior chamber"))) AND
2. (TI=("precision medicine" or "precision health" or "personalized medicine" or "personalized proteomics" or thera$nostic* or "tailored medicine" or "artificial intelligence" or "machine learning" or "deep learning" or algorithm$ or ((supervised or unsupervised or biased or unbiased or bayesian or hierarchical or neural or neuronal) NEAR/1 (cluster* or learning or learner$ or classifi* or network$)) or "k-nearest neighbo$r*" or "naive bayes" or (decision NEAR/1 (tree$ or forest$ or jungle$)) or "random forest$" or "gradient-boost*" or "support vector machine" or "k-means" or "association rules" or "recursive partitioning" or "discriminant analysis" or "feature selection" or ((linear or nonlinear or "non-linear" or logistic or ordinal or poisson or quantile or analysis) NEAR/1 (regression$ or model$)) or bioinformatic$ or ((progress* or regress* or recover* or respond* or response*) and (predict* or stratif*))) OR AB=("precision medicine" or "precision health" or "personalized medicine" or "personalized proteomics" or thera$nostic* or "tailored medicine" or "artificial intelligence" or "machine learning" or "deep learning" or algorithm$ or ((supervised or unsupervised or biased or unbiased or bayesian or hierarchical or neural or neuronal) NEAR/1 (cluster* or learning or learner$ or classifi* or network$)) or "k-nearest neighbo$r*" or "naive bayes" or (decision NEAR/1 (tree$ or forest$ or jungle$)) or "random forest$" or "gradient-boost*" or "support vector machine" or "k-means" or "association rules" or "recursive partitioning" or "discriminant analysis" or "feature selection" or ((linear or nonlinear or "non-linear" or logistic or ordinal or poisson or quantile or analysis) NEAR/1 (regression$ or model$)) or bioinformatic$ or ((progress* or regress* or recover* or respond* or response*) and (predict* or stratif*)))) AND
3. (TI=(Proteomic$ or proteome$ or metabolomic$ or metabolome$ or lipidomic$ or lipidome$ or "*inflammatory protein$" or "*inflammatory marker" or cytokine$ or interleukin$ or lymphokine$ or monokine$ or interferon$ or "colony stimulating factor$" or chemokine$ or "growth factor$" or "necrosis factor$" or "chemotactic protein$" or "adhesion molecule$" or "adhesion protein$" or "matrix metalloproteinase-2" or myeloperoxidase$ or "tissue inhibitor of metalloproteinase-2" or "macrophage inflammatory protein-1" or "brain-derived neurotrophic factor" or angiopoietin$ or ((hemoglobin or haemoglobin) NEAR/1 (a1c or glycated or glycosylated)) or hba1c or "c reactive protein" or "c-reactive protein" or crp or hscrp or "hs-crp" or ((protein or biomarker) and (concentration$ or level$ or quantif* or quantit* or mass spectrometry or iTRAQ or MALDI or SELDI or assay))) OR AB=(Proteomic$ or proteome$ or metabolomic$ or metabolome$ or lipidomic$ or lipidome$ or "inflammatory protein$" or "inflammatory marker" or cytokine$ or interleukin$ or lymphokine$ or monokine$ or interferon$ or "colony stimulating factor$" or chemokine$ or "growth factor$" or "necrosis factor$" or "chemotactic protein$" or "adhesion molecule$" or "adhesion protein$" or "matrix metalloproteinase-2" or myeloperoxidase$ or "tissue inhibitor of metalloproteinase-2" or "macrophage inflammatory protein-1" or "brain-derived neurotrophic factor" or angiopoietin$ or ((hemoglobin or haemoglobin) NEAR/1 (a1c or glycated or glycosylated)) or hba1c or "c reactive protein" or "c-reactive protein" or crp or hscrp or "hs-crp" or ((protein or biomarker) and (concentration$ or level$ or quantif* or quantit* or mass spectrometry or iTRAQ or MALDI or SELDI or assay))))

**Cochrane Central Register of Controlled Trials (CONTROL), Cochrane Database of Systematic Reviews**

1. ophth* or ocular or intraocular or eye* or retina* or macula* or fovea* or uvea* or sclera***** or cornea* or conjunctiva* or iris or "vitreous body" or "vitreous humo*r" or "vitreous fluid" or vitreo* or "aqueous humo*r" or "aqueous fluid" or tears or ((tear or lacrimal) NEXT fluid) or glaucoma or retinop* or retinoblastoma or uveitis or iritis or choroiditis or retinitis or chorioretinitis or conjunctivitis or endophthalmitis or cataract* or *opia or "optic atrophy" or "optic neuropathy" or vitrectomy or phacoemulsification or trabeculotomy or (paracentesis NEAR/3 "anterior chamber")
2. "precision medicine" or "precision health" or "personalized medicine" or "personalized proteomics" or thera*nostic* or "tailored medicine" or "artificial intelligence" or "machine learning" or "deep learning" or algorithm* or ((supervised or unsupervised or biased or unbiased or bayesian or hierarchical or neural or neuronal) NEXT (cluster* or learning or learner* or classifi* or network*)) or "k-nearest neighbo*r*" or "naive bayes" or (decision NEXT (tree* or forest* or jungle*)) or "random forest*" or "gradient-boost*" or "support vector machine" or "k-means" or "association rules" or "recursive partitioning" or "discriminant analysis" or "feature selection" or ((linear or nonlinear or "non-linear" or logistic or ordinal or poisson or quantile or analysis) NEXT (regression* or model*)) or bioinformatic* or ((progress* or regress* or recover* or respond* or response*) and (predict* or stratif*))
3. Proteomic* or proteome* or metabolomic* or metabolome* or lipidomic* or lipidome* or "*inflammatory protein*" or "*inflammatory marker*" or cytokine* or interleukin* or lymphokine* or monokine$ or interferon* or "colony stimulating factor*" or chemokine* or "growth factor*" or "necrosis factor*" or "chemotactic protein*" or "adhesion molecule*" or "adhesion protein*" or "matrix metalloproteinase-2" or myeloperoxidase* or "tissue inhibitor of metalloproteinase-2" or "macrophage inflammatory protein-1" or "brain-derived neurotrophic factor" or angiopoietin* or ((hemoglobin or haemoglobin) NEXT (a1c or glycated or glycosylated)) or hba1c or "c reactive protein" or "c-reactive protein" or crp or hscrp or "hs-crp" or ((protein or biomarker) and (concentration* or level* or quantif* or quantit* or mass spectrometry or iTRAQ or MALDI or SELDI or assay))
4. MeSH descriptor: [Ophthalmology]
5. MeSH descriptor: [Eye]
6. MeSH descriptor: [Tears]
7. MeSH descriptor: [Eye Diseases]
8. MeSH descriptor: [Vitrectomy]
9. MeSH descriptor: [Phacoemulsification]
10. MeSH descriptor: [Trabeculectomy]
11. MeSH descriptor: [Precision Medicine]
12. MeSH descriptor: [Theranostic Nanomedicine]
13. MeSH descriptor: [Artificial Intelligence]
14. MeSH descriptor: [Algorithms]
15. MeSH descriptor: [Neural Networks, Computer]
16. MeSH descriptor: [Decision Trees]
17. MeSH descriptor: [Regression Analysis]
18. MeSH descriptor: [Discriminant Analysis]
19. MeSH descriptor: [Proteomics]
20. MeSH descriptor: [Metabolomics]
21. MeSH descriptor: [Proteins] explode
22. MeSH descriptor: [Peptides]
23. MeSH descriptor: [Biomarkers]
24. concentration* or level* or quantif* or quantit* or mass spectrometry or iTRAQ or MALDI or SELDI or assay
25. (#21 or #22 or #23) and #24
26. MeSH descriptor: [Cytokines]
27. MeSH descriptor: [Cytokines]
28. MeSH descriptor: [Matrix Metalloproteinase 2]
29. MeSH descriptor: [Peroxidase]
30. MeSH descriptor: [Tissue Inhibitor of Metalloproteinase-1]
31. MeSH descriptor: [Tissue Inhibitor of Metalloproteinase-2]
32. MeSH descriptor: [Brain-Derived Neurotrophic Factor
33. MeSH descriptor: [Angiopoietins]
34. MeSH descriptor: [Anterior Eye Segment]
35. MeSH descriptor: [Anterior Capsule of the Lens]
36. MeSH descriptor: [Axial Length, Eye]
37. MeSH descriptor: [Pigment Epithelium of Eye]
38. MeSH descriptor: [Posterior Eye Segment]
39. MeSH descriptor: [Retina]
40. MeSH descriptor: [Sclera]
41. MeSH descriptor: [Tenon Capsule]
42. MeSH descriptor: [Uvea]
43. MeSH descriptor: [Asthenopia]
44. MeSH descriptor: [Cogan Syndrome]
45. MeSH descriptor: [Conjunctival Diseases]
46. MeSH descriptor: [Corneal Diseases]
47. MeSH descriptor: [Eye Abnormalities]
48. MeSH descriptor: [Eye Diseases, Hereditary]
49. MeSH descriptor: [Eye Hemorrhage]
50. MeSH descriptor: [Eye Infections]
51. MeSH descriptor: [Eye Injuries]
52. MeSH descriptor: [Eye Manifestations]
53. MeSH descriptor: [Eye Neoplasms]
54. MeSH descriptor: [Lens Diseases]
55. MeSH descriptor: [Ocular Hypertension]
56. MeSH descriptor: [Ocular Hypotension]
57. MeSH descriptor: [Optic Nerve Diseases]
58. MeSH descriptor: [Pupil Disorders]
59. MeSH descriptor: [Refractive Errors]
60. MeSH descriptor: [Retinal Diseases]
61. MeSH descriptor: [Scleral Diseases]
62. MeSH descriptor: [Uveal Diseases]
63. MeSH descriptor: [Vision Disorders]
64. MeSH descriptor: [Vitreous Detachment]
65. #1 or #4 or #5 or #6 or #7 or #8 or #9 or #10 or #34 or #35 or #36 or #37 or #38 or #39 or #40 or #41 or #42 or #43 or #44 or #45 or #46 or #47 or #48 or #49 or #50 or #51 or #52 or #53 or #54 or #55 or #56 or #57 or #58 or #59 or #60 or #61 or #62 or #63 or #64
66. #2 or #11 or #12 or #13 or #14 or #15 or #16 or #17 or #18
67. #3 or #19 or #20 or #25 or #26 or #27 or #28 or #29 or #30 or #31 or #32 or #33
68. #65 and #66 and #67
