# Supplementary material for: The role of artificial intelligence in analysis of biofluid markers for diagnosis and management of glaucoma: A systematic review
Source: Eur J Ophthalmol. 2022 Nov 25;33(5):1816–33. doi: 10.1177/11206721221140948 (PMC10469503; doi:10.1177/11206721221140948)
Supplement: sj-docx-2-ejo-10.1177_11206721221140948 - Supplemental material for The role of artificial intelligence in analysis of biofluid markers for diagnosis and management of glaucoma: A systematic review [file sj-docx-2-ejo-10.1177_11206721221140948.docx]

**Supplemental** **Materials 2**. Objectives, AI rationale, conclusions, and patient characteristics of included studies.

| First Author, Publication year | Study Objectives | Rationale for Methodology Choice | Conclusion | Mean age ± SD | Sex (male/female) | Ocular comorbidities | Systemic comorbidities | Additional reported patient characteristics (e.g. ethnicity, medications) |
| --- | --- | --- | --- | --- | --- | --- | --- | --- |
| Adav^1^, 2019 | Explore pathogenesis of PACG | Used to obtain insight into the cellular pathways most  likely impacted by proteome changes. | Altered AH proteome in human PACG implicates  oxidative stress in the neuronal damage that  preceded vision loss. | Control: 70.7±10.0  PACG: 71.0±7.0 | Control: 2/1  PACG: 0/2 | PACG (2) , cataract (3) | Not detailed | Chinese patients |
| Anton Apreutesei^2^, 2018 | Predict ocular changes in patients with diabetes and POAG | Feed forward networks can strongly predict test sets. JEN networks have high stability and can make accurate predictions because of back loops. | ANN was accurate in prediction and have demonstrated the possibility of use in predicting  the relationship between glaucoma and diabetes in a clinical environment. | 64.85±7.04 | 13/39 | POAG (all), diabetic retinopathy (6) | Diabetes (all) | Not detailed |
| Barbosa Breda^3^, 2020 | Differentiate between POAG, controls, NTG | AI algorithms were used to building classifiers that discriminated between groups. Two different classification algorithms were used and compared. | Glaucoma had higher betaine and taurine, possibly linked to neuroprotective mechanisms, and glutamate, which can indicate damaged neurons and oxidative stress. Further aqueous humor metabolomics based on NMR spectroscopy can distinguish glaucoma patients from controls with high accuracy. | POAG: 72.±10  NTG: 70±9  All glaucoma: 71±10  Control 75±8 | POAG: 10/17  NTG: 6/21  Glaucoma: 16/38  Control: 13/16 | In order of POAG, NTG, glaucoma, control:  Current trabeculectomy (11/23/0/0), current XEN (13/2/0/0), current phacoemulsification (3/2/0/0), previous phacoemulsification (8/6/0/0), previous laser capsulotomy (3/2/0/0), laser trabeculectomy (3/0/0/0) | In order of POAG, NTG, glaucoma, control:  Arterial hypertension (13/11/24/15), hyperlipidemia (9/7/16/11), heart surgery (3/1/4/3), transient ischemic attack (2/2/4/0), migraine (3/2/5/1), cancer (1/5/6/6), psoriasis (2/1/3/4), rheumatoid arthritis (3/1/4/3), thyroid disease (1/4/5/5) | Medications, in order of POAG, NTG, glaucoma, control:  IOP lowering (27/26/0/0), PGA (25/23/0/0), β-blocker (3/1/0/0), CAI (1/1/0/0), a-agonist (2/2/0/0) |
| Beutgen^4^, 2019 | Support early detection of POAG by identifying trabecular meshwork autoantibodies biomarkers | Application of ANN improves the value of biomarkers. | ANN was able to classify subjects with a sensitivity of 81% and a specificity of 93%. Discovered biomarkers are potential autoantibody biomarkers for utilization in glaucoma diagnostics. | Discovery phase - Controls: 66.17±10.22  Cases: 67±11.45  Validation phase-  Case: 62.75±12.20  Control: 63.31±15.32 | Discovery phase  POAG: 3/3  Control: 3/3  Validation phase  POAG: 30/30  Control: 23/22 | 25 control group subjects with unspecified eye disease in the, none in POAG group.  6 unspecified participants had both ocular and systemic disease. | 5 control group subjects with unspecified systemic disease in the, 0 in POAG group.  6 unspecified participants had both ocular and systemic disease. | Not detailed |
| Beutgen^5^, 2021 | Profile ocular antigens in different glaucoma subtypes | To gain insight into the characteristics of  previously identified glaucoma-related antigens and their role in glaucoma pathogenesis | The detection of significantly altered autoantibody levels  promotes prospective diagnostic glaucoma-subtyping. | Controls: 66.96 (34-83)  NTG: 68.29 (49-80)  PXG: 70.47 (52-86)  POAG: 66.37 (24-81) | Controls: 27/19  NTG: 15/16  PXG: 21/24  POAG: 25/18 | Not detailed | Not detailed | Not detailed |
| Buisset^6^, 2019 | Profile the aqueous humour in POAG patients | PCA was used to detect similar groups of samples and outliers, PLS-DA was used to discriminate between POAG and controls given metabolic profiles. | There are changes in metabolites involved in osmoprotection, neuroprotection, amino acid metabolism, remodeling of cell  membranes drained by the aqueous humor. | Control: 74.92  Case: 74.69 | Control: 15/11  Case: 15/11 | None | Diabetes, hypertension, hyperlipidemia, thyroid disease, CKD (all with no significant difference between groups) | Medications with no significant difference between groups (anti-hypertensives, lipid-lowering, antiplatelets, oral diabetes, insulin, corticosteroids, thyroid hormones, estrogen, Vit D), glaucoma medications |
| Burgess^7^, 2015 | Differentiate between POAG and controls | Clustering was used to visualize the relationship between participants and metabolites found to differentiate POAG from controls. Bioinformatics were used to translate data into defined pathways. | The specific metabolic processes highlighted may contribute to POAG status. | Control: 68.5  POAG: 67.8 | Control: 27/45  POAG: 31/41 | Not detailed | (% POAG group, % control group)  Coronary artery disease (9.7, 15.3), hypertension (54.2, 55.6), hyperlipidemia (50, 59.7), diabetes (19.4, 33.3), renal disease (0, 2.8), thyroid disease (19.4, 15.3) | Caucasian (88.9, 97.2), smokers (5.6, 6.9)  (% POAG group, % control group) |
| Csosz^8^, 2019 | Evaluate inflammatory processes on wound-healing complications after trabeculectomy | Clustering used to group significant biomarkers, bioinformatics to link biomarkers to pathways. | Inflammation has a role in wound-healing complications and PEA has utility in tear analysis. | Not detailed | Not detailed | Not detailed | Not detailed | Not detailed |
| Goto^9^, 2013 | To investigate the risk factors for NVG after vitrectomy in eyes with proliferative diabetic retinopathy | Determined risk factors and relative risks. | Risk of NVG development is associated with sex, age, IOP, preoperative neovascularization, NVG in fellow eye. | NVG: 52.7±8.8  Without NVG: 54.8±10.8 | NVG: 48/12  Without NVG: 140/54 | (# NVG group, # without NVG group)  History of panretinal photocoagulation (134/41), phakic before neovascularization (169/53), tractional retinal detachment (40/22), combined tractional retinal detachment (11/2), macular edema (25/12), vitreous hemorrhage (142/33), lens extraction (157/51), pseudophakic after vitrectomy (182/58), retinal tamponade (82/34), vitrectomy guage using a 20G (42/11), intravitreal bevacizumab preoperation (27/9), intravitreal bevacizumab immediately after vitrectomy (43/3), repeat surgery (18/7) | Not detailed | Insulin with NVG: 52  Insulin without NVG: 22  Hemodialysis (3 in NVG group) |
| Grus^10^, 2008 | Compare protein patterns in aqueous humour of POAG and control | DA selected for proteins that would discriminate between groups, ANN classified patients based on proteomic profile. | ANN could discriminate between glaucoma and control with a sensitivity of 90% and specificity of 87%. Transthyretin may be implicated in glaucoma development. | Part 1 (SELDI-TOF-MS) - POAG: 66.4±14, control: 70.5±13  Part 2 (2D electrophoresis) - POAG: 72.6±10, control: 71.8±10" | Not detailed | POAG (52), cataract (55) | Not detailed | Not detailed |
| Hennis^11^, 2003 | Examine relationship between diabetes, hypertension, and increased IOP | Examine factors associated with IOP changes between baseline and the 4-year follow-up study visit. | Increased risk of elevated IOP in populations with high prevalence of diabetes and hypertension. | 57.5 ± 11.5  Non-Participants: 60.4 ±12.4 | Participants: 436/1991 | Not detailed | Type 2 diabetes, hypertension | Residents of Barbados (all) |
| Hysi^12^, 2019 | Investigate the relationship between ascorbic acid metabolites and IOP | RF identified metabolites influential over an outcome, regardless model through which the effect is mediated. MR validated findings and assessed causality. | O-methylascorbate, has a significant IOP-lowering effect. | TWINS UK: 55±9.13  UK Biobank: 54.4±7.8  EPIC-Norfolk: 68.8±8 | TWINS UK: 8/1755  UK Biobank: 48279/55103  EPIC-Norfolk: 3059/3725 | Not detailed | Not detailed | Not detailed |
| Igarashi^13^, 2021 | Differentiate between different glaucoma subtypes using aqueous autotaxin and TGF-β | Each AI used independently to test diagnostic performance, with comparison between models. | Aqueous TGF-β and ATX exhibited high diagnostic performance as biomarkers to detect glaucoma subtypes. | No glaucoma: 72.2±9.7, POAG: 68.4±11.3, SOAG: 61.0±12.1, PXG: 75.6±9.9 | No glaucoma: 40/48, POAG: 52/45, SOAG: 32/16, XFG: 31/17 | Cataract (88), POAG (97), SOAG (48), XFG (48) | Not detailed | Not detailed |
| Iomdina^14^, 2021 | Identify changes in scleral proteins associated with POAG at different stages and IOPs | Used to determine biologic process, molecular function, and cellular component. | Scleral proteome changes indicate changes to extracellular matrix, protein glycosylation, calcium binding, fibroblast cytoskeleton regulation, oxidative or inflammatory responses. | POAG: 67.16±8.83  Control: 46.55±10.21 | POAG: 32/35  Control: 5/4 | Myopia (13.4% in cases), hyperopia (3% in cases) | Arterial hypertension (70.1% in cases, 55.5% in controls), coronary artery disease (46.3% in cases, 55.6% in controls), diabetes mellitus (10.4% cases) | Not detailed |
| Joachim^15^, 2005 | Compare IgG antibody patterns of glaucoma patients to controls | Detect differences in the distribution of antibodies against retinal, optic nerve, and optic nerve head antigen. | Highly specific autoantibodies in glaucoma subtype could provide information about pathogenic mechanism. | POAG: 67±14  NTG: 59.6±14  Control: 68.2±11 | Not detailed, gender matched | Not detailed | Not detailed | Not detailed |
| Joachim^16^, 2007 | Compare IgG antibody patterns of glaucoma patients to controls | Detect differences  in the distribution of antibodies against retinal antigens in aqueous humor of all three groups | Analysis of intraocular antibodies could provide insight into autoimmune involvement in  glaucoma. | POAG: 75.6±7  PXG: 79.9±6  Control: 68.9±13 | Not detailed, gender matched | Not detailed | Not detailed | Not detailed |
| Kouassi Nzoughet^17^, 2020 | Investigate POAG pathophysiology | Complex data mining strategy with 5 steps designed to combine the filtering method with threshold criterion, the wrapper method with iterative selection, and the embedded method with penalization constraint. | Significant findings could enable developments in the diagnosis and treatment of POAG. | POAG: 73.06 [range 61–86]  Control: 73.77 [range 57–83] | POAG: 17/17  Control: 15/15 | Not detailed | Hypertension, hyperlipidemia, thyroid disease | Not detailed |
| Lee^18^, 2017 | Investigate associations between endothelin-1, macrophage chemoattractant protein-1 levels and visual field progression in NTG | Determined factors associated with visual field progression. | Systemic MCP-1 levels associated with visual field progression in NTG patients. | 52.5±11.8 | 27/44 | Cataracts (number not detailed) | None | Glaucoma drops (number not detailed) |
| Li^19^, 2020 | Investigate association between sex hormones, inflammatory cytokines, baseline 17-b-estradiol (E2), interleukin-8 (IL-8) and visual field progression in postmenopausal women with PACG | Analyze association between sex hormones and inflammatory cytokines with PACG progression. | Decreased E2 and increased IL-8 levels are predictors of visual field progression in postmenopausal women with PACG. | No visual field progression: 66.51±6.89, visual field progression: 66.88±7.04 | All women | PACG (200 in cross-sectional, 105 in cohort) | Cross-sectional: Diabetes (13 control, 15 PACG), hypertension (42 control, 48 PACG)  Cohort: Diabetes (9), hypertension (33) | Cross-sectional: Postmenopausal (all), 1+ topical glaucoma medications (195)  Cohort: Postmenopausal (all), 1+ glaucoma medication (104) |
| Li^20^, 2020 | Investigate oxidative stress markers and visual field progression in PACG | Analyze association between oxidative stress-related factors with PACG progression. | Oxidative stress may be involved in the onset and development of PACG. | No visual field progression: 56.00±14.32, visual field progression: 61.07±12.0 | Cross-sectional -  Cases: 34/55, PACG: 37/57  Cohort - No progression: 21/30, progression: 16/27 | Cross-sectional: PACG (94)  Cohort: PACG (94) | Not detailed | Cross-sectional -Smoking (20 control, 23 PACG), drinking (22 control, 24 PACG)  Cohort – Smoking (10 in no progression, 13 in progression: 13/30), drinking (24 in no progression, 9 in progression) |
| Liang^21^, 2019 | Investigate variables associated with development of NVG after PDR vitrectomy surgery | Determined factors associated with NVG development after PPV in PDR. | Preoperative high IOP with RVO, PDR, no postoperative  intravitreal injection of ranibizumab, and higher HbA1c levels are predictors of NVG after PPV. | 55.76±10.1 | 103/135 | Mild retinopathy (159), moderate retinopathy (18), severe retinopathy (61) | Insulin-dependant diabetes (28), non-insulin-dependant diabetes (210) | Intravitreal injection ranibizumab (186) |
| Liu^22^, 2021 | Investigate of the pathogenesis of POAG | To determine cellular components, molecular functions and biological processes of all differentially expressed proteins. | GSTP1, a redox-related marker, may be involved in the pathological process of POAG and may become a treatment target in the future. | POAG: 72.80±2.60  Control: 71.70±2.51 | POAG: 8/2  Cataract: 8/2 | Cataract (all), POAG (10) | Not detailed | Not detailed |
| Myer^23^, 2020 | Determine metabolites unique to PXG when compared to POAG | PCA and PLS-DA were used to detect similar groups of samples and outliers and discriminate between conditions. 3 complex AI models were used to classify test datasets based on AH metabolome. | AI accurately classified the test datasets, and potential protein/gene pathways were associated with metabolic changes in PXG. | Not detailed | Not detailed | Not detailed | Not detailed | Not detailed |
| Nusinovici^24^, 2020 | Use machine learning to determine the contributions of factors on major eye disease development | LASSO reduced the total number of variables investigated, GBM investigated the relationships between risk factors and eye disease (accounts for the interactions and non-linear effects, accommodates for missing data) | Machine learning is useful to rank a large number of risk factors and identify the highest contributors. | 57.7±10.4 | 4946/5087 | None (4827), diabetic retinopathy (666), non-diabetic retinopathy (149), early AMD (586), late AMD (60), nuclear cataract (1518), cortical cataract (2455), posterior subcapsular cataract (1115), POAG (210), PACG (63) | Not detailed | Multi-ethnic (Chinese, Indian, Malay), smoking, alcohol, educational level |
| Pan^25^, 2020 | Identify aqueous humor metabolic biomarkers and alterations in POAG and cataract | PCA showed distribution of origin data, OPLS-DA was used to obtain a higher level of group separation and better understand variables responsible for classification, KEGG translated results into pathways of metabolites. | Identified metabolic biomarkers and pathways that may facilitate an improved understanding of the POAG pathogenesis. | POAG: 73.06 [range 61–86]  Control: 73.77 [range 57–83] | POAG: 7/9  Control: 9/15 | Not detailed | Not detailed | Not detailed |
| Park^26^, 2012 | Determine the levels of vascular endothelial growth factor in POAG, and the associations with glaucoma surgery outcomes | Assess the relationship between the clinical data and VEGF. | VEGF level was significantly related to outcomes of glaucoma surgery and associated with IOP in patients with POAG. | POAG: 58.10±12.80  Cataract: 55.64±15.56 | POAG: 8/11  Control: 10/7 | Not detailed | Diabetes (3 in POAG group, 2 in control group) | Medications used in POAG group: a-agonist (18), β-blocker (15), prostaglandins (17), carbonic anhydrase inhibitors (16) |
| Qin^27^, 2022 | Determine the association between plasma free fatty acid levels and PACG | PCA and PLS-DA performed to generate free fatty acid models to distinguish two groups. Regressions used to estimate the odds ratios. | Decreased in free fatty acid levels may be related to lipid peroxidation, and  docosahexaenoic acid and total saturated fatty acids could be used for PACG screening. | PACG: 67.57 (61.11–73.84)  Control: 69.03 (63.00–76.40) | PACG: 79/102  Control: 140/200 | Controls did not have any type of secondary glaucoma, ocular surgery within 30 days of enrollment, active uveitis, intraocular infection, retinal disease. | Hypertension (64 in PACG, 171 in control), | Current smokers (26 in PACG, 30 in control), alcohol use (10 in PACG, 14 in control), glaucoma medications (PACG group) |
| Sakamoto^28^, 2018 | Investigate variables before, during, and after vitrectomy associated with the requirement for filtration surgery | Not detailed. | Hyperglycemia, high IOP, gas tamponade during vitrectomy predisposes patients to require filtration surgery in postoperative NVG. | 52.4±9.1 | Filtration surgery needed: 34/6  No surgery needed: 15/6 | NVG (all) | Diabetes (all) | Not detailed |
| Sharma^29^, 2018 | Identify proteomic alterations in POAG | Regression adjusted for important covariates, bioinformatics was used to identify the biological processes and pathways affected by the altered proteins. | Proteins identified are implicated in signaling, glycosylation, immune  response, molecular transport, and lipid metabolism. These biomarkers may provide insight into POAH pathogenesis. | POAG: 65.1 ± 14.3  Controls: 65.8 ± 9.1 | POAG: 4/11  Controls: 12/20 | None | POAG: hypertension (12), cardiovascular disease (1), cerebrovascular disease (0), collagen vascular disease (0), diabetes (5)  Controls: hypertension (30), cardiovascular disease (4), cerebrovascular disease (3), collagen vascular disease (0), diabetes (13)" | POAG: BMI (32.2 ± 7.8), African American (8), Caucasian (7), smokers (2)  Controls: BMI (29.8 ± 5.5), African American (15), Caucasian (17), smokers (4) |
| Takayama^30^, 2019 | Determine risk factors of NVG for PDR with vitreous hemorrhage | Identify prognostic factors for postoperative NVG after vitrectomy. | Age, uncontrolled diabetes, no PRP, postoperative VH are risk factors of post-vitrectomy NVG. | Developed NVG: 50.8±12.4  Did not develop NVG: 56.1±13.0 | Developed NVG: 20/5  Did not develop NVG: 165/75 | Proliferative diabetic retinopathy (all), vitreous hemorrhage (all), tractional retinal detachment (99), pan-retinal photocoagulation ( 183) | Hypertension (189) | Anticoagulation (42) |
| Tang^31^, 2021 | Explore diagnostic, pathogenic, and therapeutic strategies in POAG patients | PLS-DA used to determine the differentially expressed metabolites in the study. | Identified biomarkers have clinical implications given that no biomarkers are currently available for glaucoma in the clinic. | POAG: 58.89 ± 14.9  Controls: 65.60 ± 11.32 | POAG: 57%/43%  Controls: 42%/58% | Not detailed | Not detailed | POAG: BMI (23.64 ± 2.08); undergoing surgeries: trabeculectomy (46%), drainage implant surgery (29%), non-penetrating trabeculectomy (11%), phacoemulsification and intraocular lens implantation (14%); glaucoma medication: Travoprost (50%), Brimonidine Tartrate (50%), Brinzolamide (46.43%), Brinzolamide and Timolol (35.71%), Bimatoprost (32.14), Timolol (10.71%), Carteolol (10.71%), Mannitol (7.14%), Latanoprost (3.57%), Tafluprost (3.57%)  Controls: BMI (25.12 ± 4.96), undergoing surgeries: phacoemulsification and intraocular lens implantation (100%)" |
| Tokuda^32^, 2012 | Predict POAG diagnosis using machine learning methods and random sampling | Complex AI pathway to classify samples as cases or controls and integrate cytokine and genetic data. | Integration of genotype and cytokine data was  effective in diagnostic prediction of POAG. | Training phase -  POAG: 56.4±5.5  Control: 55.3±3.4  Test phase -  POAH: 70.9±10.7  Control: 61.8±11.3 | Training phase -  POAG: 21/21  CTRL: 23/19  Test phase-  POAH: 45/28  CTRL: 33/19" | Not detailed | Not detailed | Not detailed |
| Voogd^33^, 2006 | Evaluate atherosclerosis and C-reactive protein as risk factors for POAG and SOAG | Compare baseline characteristics of groups and adjust for age and sex. | CRP is not a risk factor for OAG. | Participants: 65.7±6.9  Non-participants: 71.2±8.7  Died: 77.4±9.1 | Participants: 1629/2213 | Not detailed | Diabetes (6.9%), hypertension (28.9%), stroke (1.3%), dementia (0.2%) | Not detailed |
| Wakabayashi^34^, 2012 | Investigate vascular endothelial growth factor as a predictor of postoperative complications after vitrectomy for PDR (including NVG) | Determined risk factors related to postoperative complications. | High intraocular VEGF at the time of primary vitrectomy in patients with PDR is a risk factor for postoperative VH. | 58 [range 27-84) | 36/16 | Retinal detachment (19), firobascular proliferation (31), neovascularization of the disk (33), phakia (53), psuedophakia (7) | Hypertension (33), hyperlipidemia (15) | Not detailed |
| Wang^35^, 2019 | Demonstrate pathophysiology, identify biomarkers, and develop treatment strategies in PSS | PCA used to obtain an overview of the variance of metabolic phenotypes among  different groups, OPLS-DA  obtained information about the variance of metabolic phenotypes that correspond to the classes, KEGG identified relevant pathways, regression evaluated diagnostic capability of selected metabolites. | Important biomarkers could lead to better understanding of the PSS pathogenesis, and the development of novel therapeutic strategies to restrict the development/progression of PSS. | PSS: 51.3 ± 9.9  Cataract: 58.3 ± 7.9 | PSS: 5/7  Control: 4/8 | Not detailed | Not detailed | Not detailed |
| Yildirim^36^, 2008 | Determine the relationship between steroid-induced IOP rise, plasma matrix metalloproteinase-9 level, tissue inhibitor of MMP-2 in diabetic patients who underwent intravitreal  triamcinolone acetonide injection for DME treatment | Determine effect of plasma levels of MMP-9, TIMP-2 on IOP. | Elevated TIMP in diabetic patients might have a role on steroid-induced IOP rise. | Diabetic macular edema: 57.6±10.2  Control: 53.1 ± 10.3 | Diabetic macular edema: 23/14  Control: 10/7 | Diabetic macular edema (cases) | Diabetes (cases) | Topical anti-glaucomatous medication (all) |
| Zhang^37^, 2018 | Quantify matricellular proteins in the  aqueous humor in patients with previous PACG and investigate  their relationship with bleb morphology and trabeculectomy outcomes. | Binary logistic regressions used to assess relationship between the clinical outcomes and matricellular protein levels. | Secreted protein acidic and rich in cysteine level in aqueous humor is a prognostic factor for surgical results of trabeculectomy. | 68±11 | 15/25 | Not detailed | Not detailed | Topical  Pilocarpine, topical b-adrenergic  Antagonists, topical a2-adrenergic agonists, topical steroids,  topical and/or oral carbonic anhydrase inhibitors |
| Zhavoronkov^38^, 2016 | Explore mechanisms that explain clinical cases of POAG | Pathway Activation Strength allowed quantitative measure of differential pathway activation between the 2 states. | The pathways uncovered have potential therapeutic utility to delay or avoid fibrosis initiation in TM and LC tissues. | Not detailed | Not detailed | Not detailed | Not detailed | Not detailed |
| Zhu^39^, 2019 | Evaluate thrombospondin-1 as a predictor of prognosis of trabeculectomy in PACG | Assessed potential risk factors for failure of surgery. | TSP-1 and TGF-β2 levels are higher in PACG eyes with failed trabeculectomy at one year. | Case: 56.53±9.32  Control: 56.55±10.29 | Case: 7/19  Control: 22/56 | Case: combined phacoemulsification (9), shallow anterior chamber (9)  Controls: combined phacoemulsification (15), shallow anterior chamber (7) | Case: diabetes (5), hypertension (6)  Controls: diabetes (13), hypertension (27) | Han Chinese participants,  preoperative mean glaucoma medication (case = 2.92±0.69, control = 3.09±0.54) |

**Acronyms**: AI = artificial intelligence, AMD = age related macular degeneration, ANN = artificial neural network, CKD = chronic kidney disease, DA = discriminant analysis, DME = diabetic macular edema, DR = diabetic retinopathy, EPIC-Norfolk = European Prospective Investigation into Cancer – Norfolk, IOP = intraocular pressure, IPA = ingenuity pathways analysis, LASSO = least absolute shrinkage and selection operator, MR = mendelian randomization, NDR = nonproliferative diabetic retinopathy, NMR = nuclear magnetic resonance, NTG = normal tension glaucoma, NTG = normal tension glaucoma, NVG = neovascular glaucoma, NVG = neovascular glaucoma, PACG = primary angle closure glaucoma, PCA = principal component analysis, PDR = proliferative diabetic retinopathy, PLS-DA = partial least-squares discriminant analysis, POAG = primary open angle glaucoma, PPV = pars plana vitrectomy, PXG = pseudoexfoliation glaucoma, RF = random forest, SOAG = secondary open angle glaucoma
