# Supplementary material for: The role of artificial intelligence in analysis of biofluid markers for diagnosis and management of glaucoma: A systematic review
Source: Eur J Ophthalmol. 2022 Nov 25;33(5):1816–33. doi: 10.1177/11206721221140948 (PMC10469503; doi:10.1177/11206721221140948)
Supplement: sj-docx-3-ejo-10.1177_11206721221140948 - Supplemental material for The role of artificial intelligence in analysis of biofluid markers for diagnosis and management of glaucoma: A systematic review [file sj-docx-3-ejo-10.1177_11206721221140948.docx]

**Supplemental** **Materials 3**. Joanna Briggs Institute Critical Appraisal Tools for included studies.


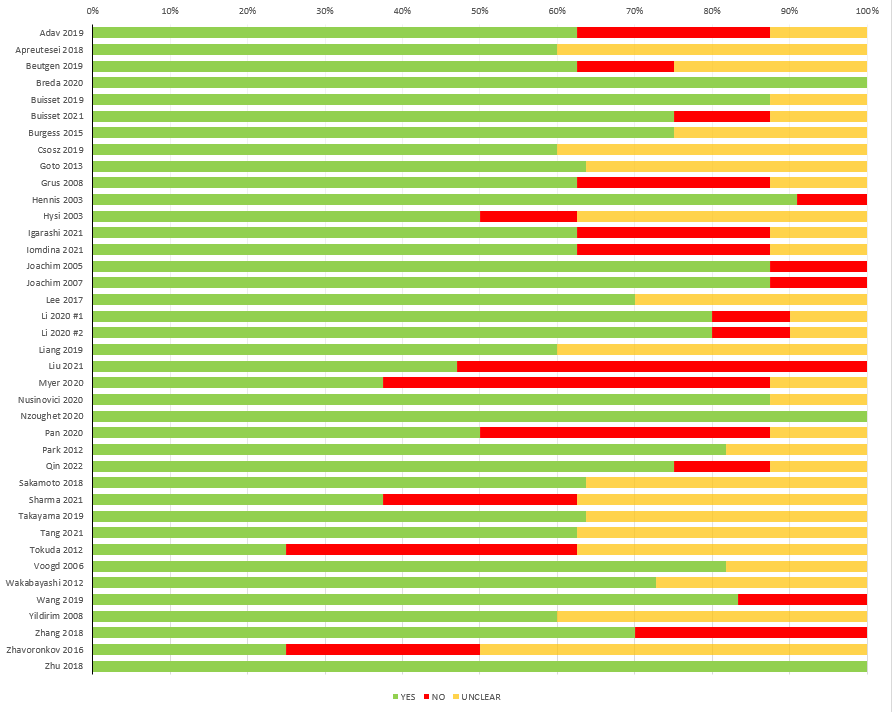


The Joanna Briggs Institute Critical Appraisal Tools provides 8-11 questions about study bias based on study type. The legend “Yes”, “No”, and “Unclear” refer to the possible answers to these questions. The response “Yes” indicates that the study successfully accounted for a risk of bias, while “No” means they did not.
